# Characterization of Immunogenicity of Malignant Cells with Stemness in Intrahepatic Cholangiocarcinoma by Single-Cell RNA Sequencing

**DOI:** 10.1155/2022/3558200

**Published:** 2022-04-29

**Authors:** Jing Bian, Jianyang Fu, Xin Wang, Jihye Lee, Gagandeep Brar, Freddy E. Escorcia, Maggie Cam, Changqing Xie

**Affiliations:** ^1^CCR Collaborative Bioinformatics Resource, National Cancer Institute, National Institutes of Health, Bethesda, Maryland, USA; ^2^Gastrointestinal Malignancies Section, Center for Cancer Research, National Cancer Institute, National Institutes of Health, Bethesda, Maryland, USA; ^3^Sandra and Edward Meyer Cancer Center, Weill Cornell Medicine, New York, New York, USA; ^4^Molecular Imaging Branch, Radiation Oncology Branch, Center for Cancer Research, National Cancer Institute, National Institutes of Health, Bethesda, Maryland, USA; ^5^NCI CCR Liver Cancer Program, USA

## Abstract

Cancer stem cells (CSCs) are responsible for long-term maintenance of tumors and thought to play a role in treatment resistance. The interaction between stemness and immunogenicity of CSCs in the intrahepatic cholangiocarcinoma (iCCA) is largely unknown. Here, we used single-cell transcriptomic data to study immunogenicity of malignant cells in human iCCA. Using an established computerized method CytoTRACE, we found significant heterogeneity in stemness/differentiation states among malignant cells. We demonstrated that the high stemness malignant cells express much lower levels of major histocompatibility complex II molecules when compared to low stemness malignant cells, suggesting a role of immune evasion in high stemness malignant cells. In addition, high stemness malignant iCCA cells exhibited significant expression of certain cytokine members, including CCL2, CCL20, CXCL1, CXCL2, CXCL6, CXCL8, TNFRSF12A, and IL6ST, indicating communication with surrounding immune cells. These results indicate that high stemness malignant cells retain their intrinsic immunological feature that facilitate the escape of immune surveillance.

## 1. Introduction

Intrahepatic cholangiocarcinoma (iCCA) is a highly lethal malignancy originating from the intrahepatic bile ducts proximal to the second-order branch division whose global incidence has been rising over the past few decades [1]. A minority of patients with early stage iCCA are eligible for curative-intent surgical resection, which is the only treatment shown to confer long-term survival. Still, recurrence rates remain high even in this setting. Most patients are diagnosed with advanced disease and have limited available therapeutic options [2]. Several studies have demonstrated modest but statistically significant survival advantage using chemotherapy [3, 4] and targeted agents [5, 6]. Although immune checkpoint inhibitors (ICI) have shown remarkable success in other cancer types, they have demonstrated modest efficacy in iCCA [7, 8]. Therefore, there remains a critical unmet need to improve our understanding of pathogenesis of iCCA to inform basis of therapeutic strategies that could improve survival for this malignancy.

Cancer stem cells (CSC) are a subpopulation of cells that exist within the tumors and are responsible for long-term maintenance of tumors by supplying renewable source of malignant cells, which is thought to contribute to treatment resistance [9–11]. It has been historically challenging to identify CSCs, although they are known to be positive for various cell surface markers, such as CD133 and CD24. CSCs are believed to remain in a quiescent state until stimulated by signals in the tumor microenvironment (TME). Once activated, CSCs can give rise to new terminally differentiated malignant cells leading to tumor initiation, metastases, and recurrence [12, 13]. Recent reports have shown a negative association between cancer stemness and antitumoral immunity, suggesting that the presence of CSCs can lead to an immunosuppressive TME partially through the interaction with surrounding stromal cells [14, 15]. There have been several efforts to characterize the immunogenicity of CSCs, although they were mainly performed using established cell lines [16–18]. The loss of certain phenotypic properties of these cell lines and variability of different antibodies to detect CSCs in the tumors can inaccurately reflect the real immunogenicity of CSCs in vivo [16, 19, 20]. As the immunological characterization of CSCs in vivo is largely unknown, more studies aimed towards direct analysis of patient data will provide better understanding of underlying role of stemness in therapy resistance.

Recent single-cell RNA sequencing (scRNA-seq) technology has enabled the large-scale profiling of transcriptomic states/stochasticity at the single-cell resolution level and provides insights into transcriptional stochasticity. CytoTRACE is a newly developed computational algorithm for predicting the differentiation status of malignant cell population based on scRNA-seq data [21–23]. Here, we used publicly available scRNA-seq data to characterize the stemness phenotype and immunogenicity of high stemness in iCCA.

## 2. Materials and Methods

### 2.1. Data Download and Preprocessing of scRNA-seq Data

Raw scRNA-seq data was downloaded from GSE125449 (9 HCC and 10 iCCA samples) [24] and GSE138709 (5 iCCA samples) [25] downloaded from Gene Expression Omnibus. These data were last updated on October 6, 2019, and June 6, 2020, respectively. Downstream analysis using Seurat and visualization was performed within the NIH Integrated Analysis Portal (NIDAP) using R programs developed on the Palantir Foundry platform (Palantir Technologies). Initial processing of single-cell data used the Seurat workflow [26]. The cells were preprocessed according to their unique molecular identifier (UMI) counts, number of expressed genes, and mitochondrial content; the cells containing fewer than 2001 UMIs, greater than 6000 expressed genes or fewer than 501 genes, were excluded from the data, along with the cells that have above 20% mitochondrial genes. The effects of UMI counts and mitochondrial content were regressed out, and the gene expression data was normalized using Seurat sctransform function [27]. Batch effects among the samples were accounted for using Harmony [28], and batch corrected gene expression matrix was backcalculated from the Harmony cell embeddings.

### 2.2. Identification of Malignant Cells from Normal Cholangiocytes with CNV

The cells were identified according to the aggregate expression of marker sets specified in [25]. Background noise was accounted for by subtracting the average expression of 100 randomly sampled genes from the aggregate result. To confirm the identities of malignant cells and cholangiocytes, we calculated the CNV of cells using infercnv [29]. For this analysis, we designated cells from adjacent samples as the reference input to infercnv. The cells with a mean cutoff value less than 1 were excluded from the analysis. A background noise filter value of 0.2 was applied.

### 2.3. CytoTRACE Analysis

CytoTRACE was used to estimate transcriptional diversity of each malignant cells in terms of differential or stemness status [21]. The cells were given a CytoTRACE score according to their differentiation potential, with a higher score indicating higher stemness/less differential characteristics. For this study, the cells with CytoTRACE scores above 0.75 were designated as high stemness malignant cells, while the cells with scores below 0.25 were designated as low stemness malignant cells.

### 2.4. Gene Enrichment Analysis

Genes with *P* value < 0.05 were filtered, and pathway analysis was performed on significantly upregulated and downregulated genes using the l2p [30] package with the following databases: KEGG, GO, Reactome, and Hallmark Genes from the Molecular Signature Database (MSigDB v6.2) [31].

In addition, gene set enrichment analysis (GSEA) was performed using fgsea R package [32]. For the gene sets, a custom curated list of genes associated with stemness [15, 33–37] was added to the Hallmark, CP:Reactome, and KEGG gene sets. The differential gene expression results between high stemness malignant cells and low stemness malignant cells were ranked according to the chi-squared statistic multiplied by the sign of the log2 fold change and used as inputs to GSEA. The *P* values associated with GSEA were adjusted according to the methods of Benjamini-Hochberg [38].

### 2.5. Identification of Immune Signature Genes in CSCs

We collected 2,341 immunology-related genes from KEGG terms “immune system” and “immunological diseases” from the KEGG website (https://www.kegg.jp/kegg/pathway.html#cellular), including MHC family, cytokine and chemokine family, nature killer cell-mediated cytotoxicity members, and TGF*β* and TNF*α* signaling pathways using the I2pgetgenes4acc function from the I2p package. The immune signature of high stemness malignant cells was compared to that of low stemness malignant cells using the Kruskal-Wallis analysis of variance. The *P* values were adjusted according to the methods of Benjamini-Hochberg.

### 2.6. Communications of Malignant Cells and T Cells

We used CellphoneDB [39] to study the ligand-receptor interactions of high stemness/low stemness malignant cells with CD4/CD8 T cells and high stemness/low stemness malignant cells with natural killer and myeloid cells. Ligand-receptor pairs with *P* value < 0.05 were considered as significant.

### 2.7. Immunofluorescence

The slides with tumor sections were fixed using freshly made 4% paraformaldehyde for 20 minutes. The slides were blocked with fetal bovine serum for an hour at room temperature before incubating in primary antibodies for 18 hours. The primary antibodies include anti-CD133 (1 : 100, Abcam, Waltham, MA), anti-TACSTD2 (1 : 100, Invitrogen, Waltham, MA), monoclonal mouse antihuman Epithelial Related Antigen Clone MOC-31 (1 : 100, Dako), CXCL8 polyclonal antibody (1 : 100, Invitrogen, Waltham, MA), CXCL1 polyclonal antibody (1 : 100, Invitrogen, Waltham, MA), Rabbit (DA1E) mAb IgG XP® isotype (1 : 100, Cell Signaling Technology, Danvers, MA), and purified mouse IgGa, kappa isotype control antibody (1 : 100, Biolegend, San Diego, CA). Secondary antibodies included goat pAb to RB IgG (1 : 500, Abcam, Waltham, MA) and goat pAb to Rb IgG (1 : 500, Abcam, Waltham, MA), and the samples were incubated for an hour in a dark environment. Diluted Hoechst was added during the last 15 minutes of the incubation. The slides were washed with PBS and mounted with ProLong™ Gold antifade reagent (Thermo Fisher, Waltham, MA). Imaging of the slides was carried out using AxioVision version 4.7.1.

### 2.8. Quantification and Statistical Analysis

Statistical analysis was performed using the Wilcox.test function in R (version 3.6.3) and GraphPad Prism (version 7.04). Wilcoxon's rank-sum test, Student's *t*-test, and Hotelling's T-squared test were used in this study.

## 3. Results

### 3.1. Transcriptomic Intratumoral Heterogeneity of Malignant Cells in iCCA

**Figure 1:**
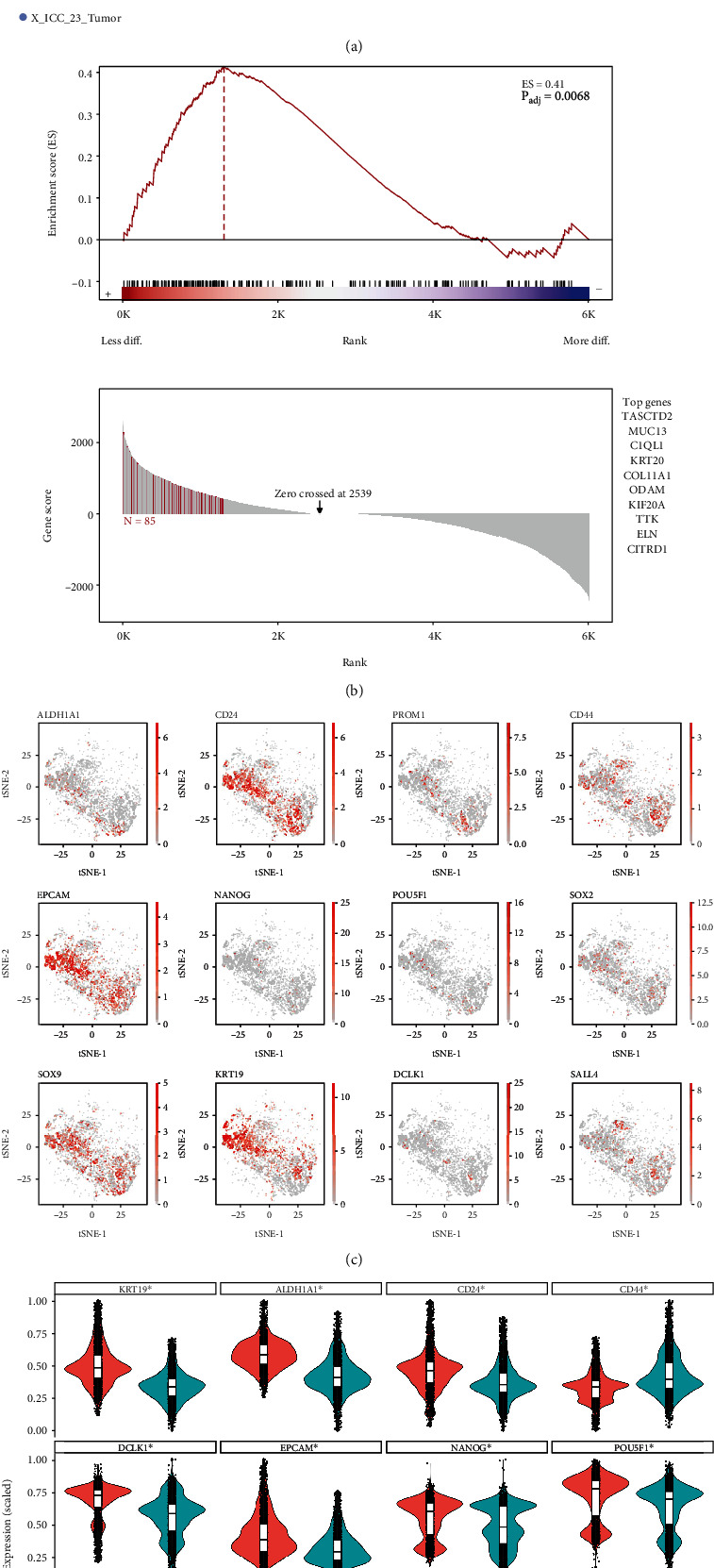
Differentiation heterogeneity of malignant cells in iCCA. (a) tSNE plots for malignant cells from 5 tumor samples (upper panel). CytoTRACE analysis of malignant cells (lower panel). CytoTRACE scores projected with tSNE plots are colored red to indicate high stemness/low differentiation and blue for low stemness/high differentiation. (b) GSEA enrichment and leading-edge plots. (Top) Differentially expressed genes found in CytoTRACE classified high stemness malignant cells and low stemness malignant cells. Genes were ranked by Log2FC. (Bottom) Genes contributing the most to the enrichment score. The top 10 genes that are predicted to be specifically associated with high stemness malignant cells are indicated on the right box. (c) tSNE plots showing the expression of CSC marker genes. (d) Violin plots showing the expression of CSC marker genes. ∗ indicates *P* < 0.05.

scRNA-seq data from biopsied iCCA tumor samples were downloaded and reprocessed using parameters matching the original publication [25]. The regenerated t-SNE plot (data not shown) were found to correspond well to the published data [25]. With the linearly uncorrelated principal components (PCs) (*k* = 10), we performed t-SNE analysis (data no shown) to visualize high-dimensional data in a two-dimensional space. These analyses correlated to the published data [25] and confirmed the reproducible visualization of the t-SNE plot. Malignant cell and cholangiocyte identities were confirmed using CNV analysis. This exercise led to a total of 11993 malignant cells for further analysis (Figure 1(a), upper panel, and Supplemental Table 1). CytoTRACE was employed to investigate the transcriptional heterogeneity and differential status/stemness level of individual malignant cells retained in this study. As shown in Figure 1(a) (bottom panel), the CytoTRACE score was diversely distributed, indicating that there was heterogeneity among tumor cells in terms of stemness/differentiation status. To validate the ability of CytoTRACE to define iCCA malignant cell differentiation states, we first analyzed differentially expressed genes (DEGs) between CytoTRACE classified high stemness malignant cell population (CytoTRACE scores above 0.75) and low stemness malignant cell population (CytoTRACE scores below 0.25). We rank-ordered the genes based on their log_2_FC values and used this ranked list to run GSEA on an independent list of genes associated with stemness in humans (Figure 1(b), upper panel, and supplemental table 2). We found significant enrichment of previously reported genes related to stemness (e.g., TACSTD2 [40, 41] and ROR1 [42], enrichment score 0.41 and *P*_adj_ = 0.0068) (Figure 1(b), lower panel). There was a considerable overlap of expression of TACSTD2 and previously reported CSC marker CD133 (PROM1) evidenced by immunofluorescence (supplemental Figure1A), which suggests that CytoTRACE is a potential platform to defining malignant cell at separate differentiation states in iCCA. Interestingly, the expression pattern of majority of reported CSC surface markers matches the CytoTRACE score distribution pattern, including ALDH1A1, CD24, EPCAM, POU5F1, SOX2, and KRT19 (Figures 1(c) and 1(d)). Meanwhile, there is no single CSC surface marker exclusively expressed in high stemness malignant cells with high CytoTRACE score, indicating the plasticity of malignant cells with stemness feature and the necessity of exploring new markers to identify true CSCs (Figure 1(c)). In addition, some of the markers that were used broadly in the previous CSC studies were not significantly expressed in high stemness malignant cells, such as CD44, indicating that there is data discrepancy derived from the in vitro and in vivo experiments.

**Figure 2:**
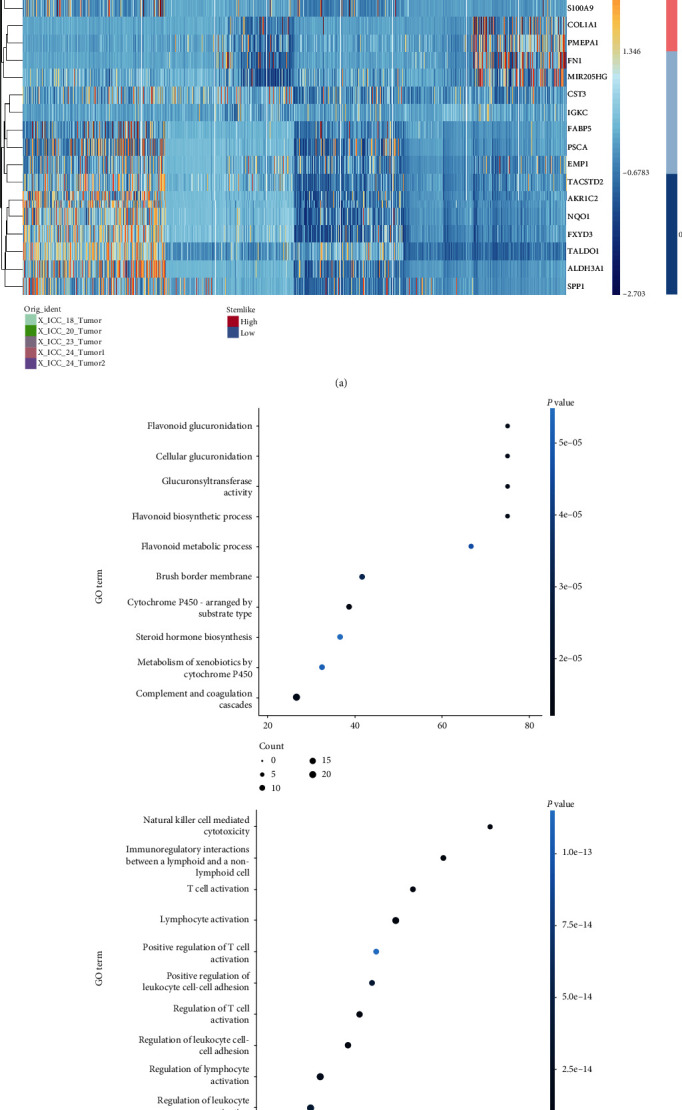
Differential expressed genes between high stemness and low stemness iCCA cells. (a) Heatmap showing top 10 DEGs (upregulated and downregulated) between high stemness and low stemness malignant cells. (b) Differences in pathway activity (scored per cell by l2p) between high and low stemness malignant cells (upper panel: upregulated genes; lower panel: downregulated genes).

We further compared the DEGs of high stemness malignant cells to the ones with low stemness. Remarkably, the results showed transcriptomic differences between high and low stemness malignant cells (Figures 2(a)–2(d) and supplemental table 3). Among these upregulated genes in high stemness malignant cells in comparison to the ones with low stemness, for example, aldehyde dehydrogenase family 3A (ALDH3A1) activity has been used as one of markers of stemness and secreted phosphoprotein 1 (SPP1), which can bind to CD44 to maintain stemness [43]. Among those downregulated genes, cytokeratin 6 and 17 (KRT6 and KRT17) and collagen members (COL4A1 and COL6A2) are related to terminal differential status of malignant cells. Gene set variation analysis of DEGs indicated that there was upregulation of genes associated with of metabolic pathway and considerable downregulation of immune-relevant pathway in the higher stemness malignant cells. These different functional patterns of malignant cells between high and low stemness likely reflect the essential activity difference of high stemness cell population versus differential malignant cells. Taken together, these findings suggest a correlation of CytoTRACE analysis with stemness status of malignant cells in iCCA. Thereafter, we use malignant cells with a high CytoTRACE score as a substitute of malignant cells with high cancer stemness feature.

We also used the other set of publicly available scRNA-seq date derived from iCCA to further validate the CytoTRACE for potential usefulness to explore CSC signature GSE125449 [24] (Supplemental Figure S2 and supplemental table 4). Although there were some differences between these two cohorts in terms of expression of reported CSC markers, expression similar pattern was generally concordant, confirming that high stemness malignant cells exhibited significant higher expression level of CSC markers, including KRT19, EPCAM, CD24, ALDH1A1, and SOX2.

### 3.2. Immunogenicity of Malignant Cells with High Cancer Stemness Feature in iCCA

**Figure 3:**
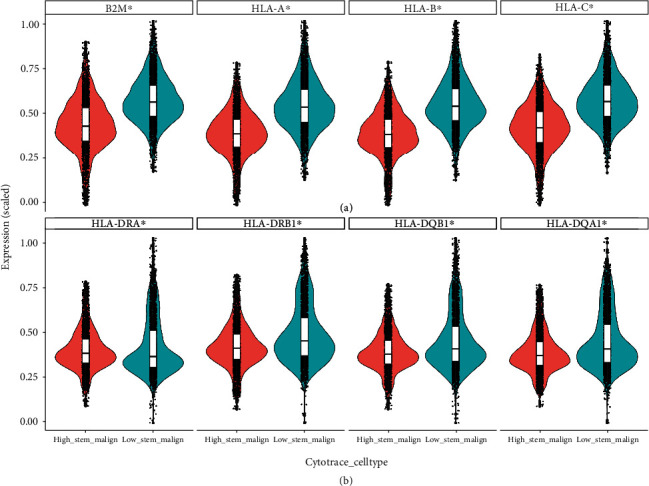
Comparison of MHC pathway profile between high stemness and low stemness iCCA cells. (a) Violin plot of MHC I pathway-related genes. (b) Violin plot of MHC II pathway-related genes. ∗ indicates *P* < 0.05.

**Figure 4:**
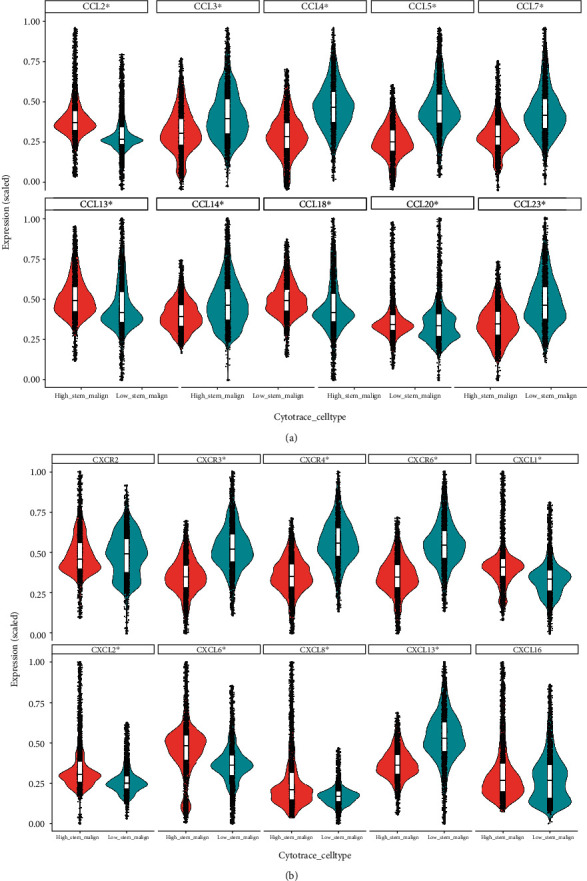
Comparison of C-C and C-X-C cytokine profile between high stemness and low stemness iCCA cells. (a) Violin plot of C-C chemokines. (b) Violin plot of C-X-C chemokines. ∗ indicates *P* < 0.05.

**Figure 5:**
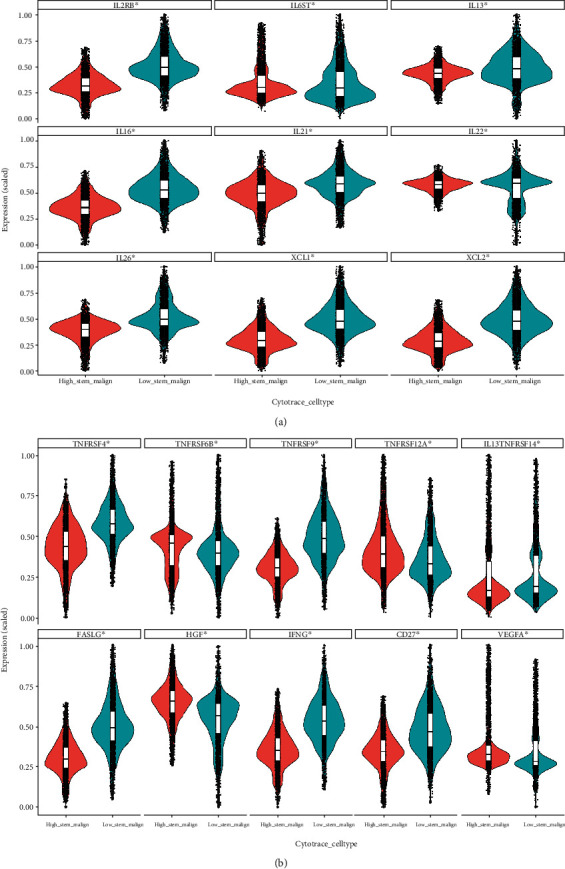
Comparison of other inflammatory factor profile between high stemness and low stemness iCCA cells. (a) Violin plot of interleukin family. (b) Violin plot of TNF family and other inflammatory factors. ∗ indicates *P* < 0.05.

To further evaluate the potential mechanism of immune evasion of high stemness malignant cells, we further analyzed the expression patterns of immune-related genes of iCCA malignant cells based on scRNA-seq data (Figures 3–5, supplemental table 3-4, and Supplemental Figure S3-S5).

The expression of major histocompatibility complex (MHC) class I and II molecules and antigen-presenting machinery (APM) is essential to display antigen peptides to cytotoxic T cells and trigger a response against non-self-antigens. As shown in Figures 3(a) and 3(b) and Supplemental Figure S3-S4, there is considerable expression of *β*2M, MHC class I and II, and transporter associated with antigen processing (TAP) molecules in all malignant cells from both cohorts. Although there is a discrepancy in expression of MHC class I between high stemness versus low stemness malignant cells from these two cohorts, there is significant lower expression of MHC class II molecules and TAP1 in high stemness malignant cell population (Figure 3 and Supplemental Figure S3-S4), which indicates that the MHC II pathway in iCCA CSCs likely contributes to immune evasion during tumor initiation and progression.

Other than critical MHC molecules, there is an enriched list of inflammatory factors which are expressed by malignant cells to conduct important communication messengers with surrounding stromal cells. In this study, we found that high stemness malignant cells expressed considerable level of inflammatory factors (presumably early on) to build up the niche for further survival from immune surveillance and eventually tumorigenesis. Furthermore, some inflammatory factors were expressed at a higher level in the high stemness malignant cells from both cohorts. These factors included CCL2 and CCL20 (Figure 4(a) and Supplemental Figure S5A), CXCL1, CXCL2, CXCL6, and CXCL8 (Figure 4(b) and Supplemental Figure S5B), and IL6ST and TNFRSF12A (Figure 5 and Supplemental S5C and S5D). There were overlaps between the expression of TACSTD2 and CXCL1 and CXCL8, evidenced by immunofluorescence (Supplemental Figure S1B-S1C). Meanwhile, some inflammatory factors were expressed at a significant lower level in the high stemness malignant cells from both of cohorts. These factors included CCL3, CCL4, CCL5, CCL13, and CCL14 (Figure 4(a) and Supplemental S5A), CXCR3, CXCR4, CXCR6, and CXCL13 (Figure 4(b) and Supplemental S5B), and IL2RB, IL16, XCL1, XCL2, TNFRSF4, IFNG, and CD27 (Figure 5 and Supplemental S5C and S5D). These suggest that certain inflammatory factors expressed in high stemness malignant cells may contribute to stemness maintenance while also facilitating immune evasion.

**Figure 6:**
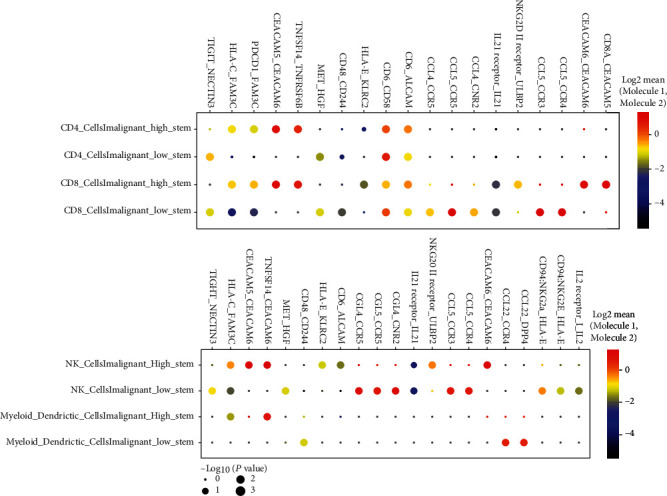
Communication between malignant cells and immune cells. Dot plot showing top predicted ligand-receptor interactions of malignant cells and T cells (upper panel) and malignant cells and NK/myeloid/dendritic cells (lower panel). Column represents ligand and receptor pairs. Red, ligand from malignant cells; blue, receptor from immune cells. Row represents pair of malignant cells (red) and immune cell subtype (blue).

Meanwhile, we used the computerized algorithm CellPhoneDB to predict ligand-receptor interaction between malignant cells and immune cells. We observed that HLA-C/FAM3C, CEACAM5/CEACAM6, and TNFSF14/TNFRSF6B were commonly enriched between high stemness malignant cells and all immune cells (Figure 6). Notably, PDCD1/FAM3C was enriched between high stemness malignant cells with T cells specifically. These results have demonstrated the complex nature of high stemness malignant cells, which greatly influence surrounding immune cell functionalities. Taken together, these interactions suggest that blocking these axes may affect the interaction of high stemness malignant cells with surrounding immune cells and could be an effective strategy to overcome therapeutic resistance for iCCAs.

## 4. Discussion

In this study, we used CytoTRACE to stratify malignant cells to different groups depending on stemness/differentiation states and further characterized immunogenicity of high stemness malignant cells. Our results indicate that heterogenicity exists within malignant cells with respect to stemness and their immunity. We found that malignant cells with high stemness express much lower levels of MHC class II and TAP1 molecules and exhibit significant expression of certain inflammatory factors with some of them much higher expressed in comparison to low stemness malignant cells. These results indicate that high stemness malignant cells retain their intrinsic immunological feature that facilitate the escape of immune surveillance. Our study represents the first report to demonstrate the immunological characteristics of high stemness malignant cells in iCCA with scRNA-seq.

It has been challenging to define CSC population in vivo given no consensus on a specific and universal signature of CSCs across tumor types, though various cell surface markers have been employed to better define CSCs in both cell lines and tumors, including CCA [44]. Here, with single-cell resolution, we found that different CSC marker-defined malignant cells have distinct transcriptomic profiling, which likely reflects phenotypic and functionally differences in plasticity and differentiation [45–47].

The mechanism of CSC immune evasion has been remaining elusive. Immune recognition of tumor antigens by cytotoxic T lymphocytes is mediated through MHC molecules on the cell surface with the assistance of APM. In this study, we demonstrated that high stemness malignant cell population from iCCA tumor samples exhibited significantly lower levels of MHC molecules compared to low stemness malignant cells. Our findings are consistent with the previous reports using in vitro culture from glioma [16], melanoma [48], and colon [49] samples, all of which have documented reduced expression of MHC. Together, these data support the hypothesis that high stemness malignant cells reduces host immune recognition and is a strategy used by malignant cells to escape from immune surveillance.

The immunological profiling studies from established human CCA cell lines have shown that CCA spheroids, a method to enrich cells with stemness status in vitro, release a spectrum of inflammatory molecules that presumably execute immunomodulatory effects on the TME [20]. Here, we found that high stemness iCCA cells express proinflammatory factors, including CCL2, CCL20, CXCL1, CXCL2, CXCL6, and CXCL8, although none of these was exclusively expressed in all high stemness malignant cells. These results differ from previously published reports using established cell lines as opposed to biopsied tumors from patients [20], which may in part explain the discordance [20]. CXCL1, 2, 6, and 8 belong to ELR (glutamic acid-leucine-arginine)-positive CXC chemokines, and it is well established that these family of chemokines are found to promote angiogenesis. These findings are consistent with the prior reports that CSCs may be a crucial source of key angiogenic factors in the early phase of tumorigenesis [50].

Recently, it was reported that both CXCL1 [51] and CXCL2 [52] are important for immune evasion through recruitment of CXCR2-positive myeloid-derived suppressor cells (MDSC). Since MDSCs can suppress effector T cell activation, proliferation, trafficking, and viability, inhibit NK cells, and activate regulatory T cells, these CXC chemokines can potentially contribute to CSC immune evasion [53]. Intriguingly, our study also showed considerable high expression levels of HGF and VEGF in some high stemness malignant cells. HGF could synergistically enhance new blood vessel generation [54], which likely facilitates survival of high stemness malignant cells initially, and suggest that antiangiogenesis in combination with immunotherapy may overcome immunotherapy resistance in iCCA. Furthermore, ligand-receptor analysis showed certain stronger interaction between high stemness malignant cells with immune cells in comparison to low stemness malignant cells, e.g., PDCD1-FAM3C pair, where FAM3C is noted to drive breast CSC formation [55], while PDCD1 expression is the marker of exhausted T cells and has a core role for tumor evasion from immune surveillance. The blockage of this interaction will likely change the communication between high stemness malignant cells and immune cells though biological function of this interactions need to be further characterized.

Together, our results support the hypothesis that high stemness iCCA cells are associated with reduction of immune recognition and expression of profound inflammatory factors, leading to the generation of an immunosuppressive TME in iCCA. Because the data analysed here are derived from scRNA-seq of patient tumors, additional confirmatory studies are warranted. Specifically, in vitro and in vivo studies are needed to formally dissect molecular mechanisms underlying interactions of high stemness malignant cells and the individual neighboring immune cell subset in the TME. Such efforts could lead to the development of novel therapies to overcome treatment resistance and improve outcomes for patients with this highly lethal malignancy.

## 5. Conclusions

CytoTRACE can be used for stratifying high stemness malignant cells from scRNA-seq data of iCCA. High stemness iCCA cells express low levels of MHC II and considerable cytokines to evade immune surveillance and concurrently generate immunosuppressive TME.

## Data Availability

scRNA-seq data can be downloaded from GSE138709 and GSE125449. Other data supporting the conclusions of this article will be made available by the authors without reservation.

## Supplementary Materials

Supplementary Materials Supplemental Table S1: malignant cell counts from 5 tumor samples. Supplemental Table S2: collection of stemness signatures from the previous publications. Supplemental Table S3: fold change of genes between high stemness and low stemness malignant cells and *P* value from GSE138709. Supplemental Table S4: fold change of genes between high stemness and low stemness malignant cells and *P* value from GSE125449. Supplemental Figure S1: representative immunofluorescence images. Bar = 37.74 *μ*m. Supplemental Figure S2: differentiation heterogeneity of malignant cells in iCCA from GSE125449. S2A: tSNE plots for malignant cells showing CytoTRACE analysis of malignant cells. S2B: tSNE plots showing the expression of CSC marker genes. S2C: violin plots showing the expression of CSC marker genes. ∗ indicates *P* < 0.05. Supplemental Figure S3: comparison of TAP1 and TAP2 between high stemness and low stemness iCCA cells from GSE138709, shown with violin plot. ∗ indicates *P* < 0.05. Supplemental Figure S4: comparison of MHC pathway profile between high stemness and low stemness iCCA cells from GSE125449. S4A: violin plot of MHC I and II pathway-related genes. S4B: violin plot of TAP1 and TAP2. ∗ indicates *P* < 0.05. Supplemental Figure S5: comparison of inflammatory factors between high stemness and low stemness iCCA cells from GSE125449. S5A: violin plot of C-C chemokines. S5B: violin plot of C-X-C chemokines. S5C: violin plot of interleukin family. S5D: TNF family and other inflammatory factors. ∗ indicates *P* < 0.05.
