# Supplementary material for: Characterization of Immunogenicity of Malignant Cells with Stemness in Intrahepatic Cholangiocarcinoma by Single-Cell RNA Sequencing
Source: Stem Cells Int. 2022 Apr 29;2022:3558200. doi: 10.1155/2022/3558200 (PMC9076354; doi:10.1155/2022/3558200)

Supplemental Figure S1

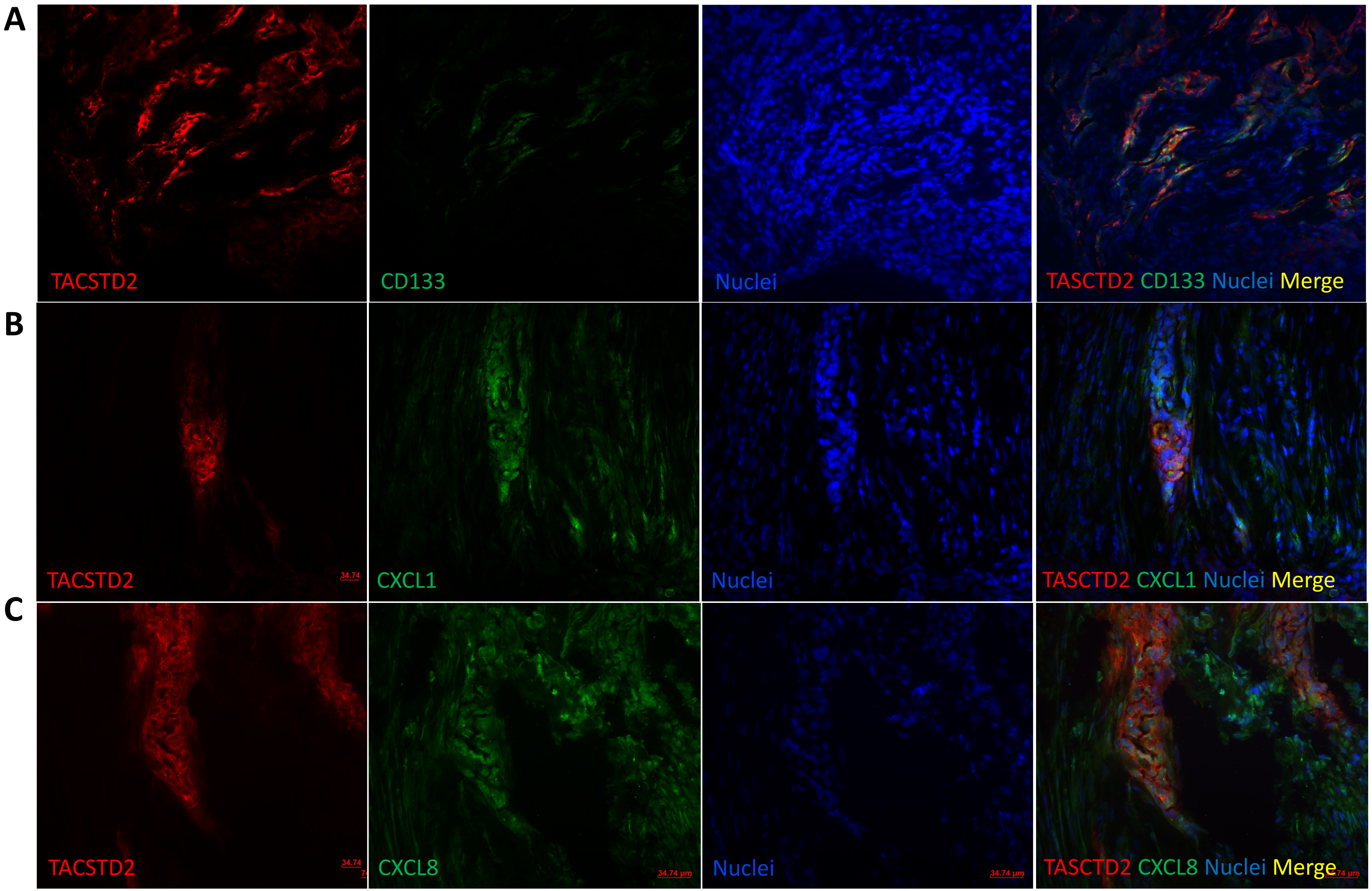

Supplemental Figure S2

S2A

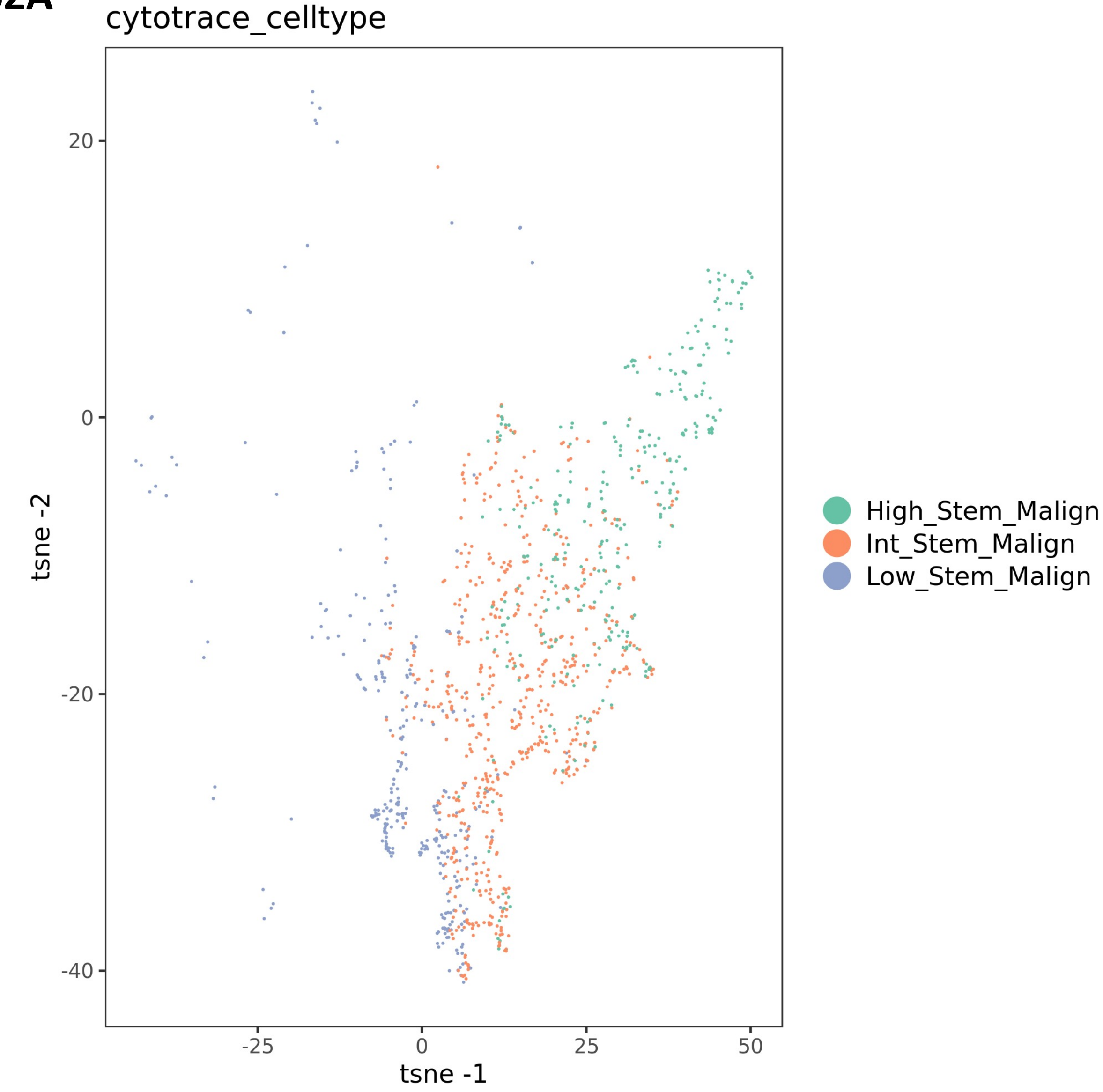

S2B

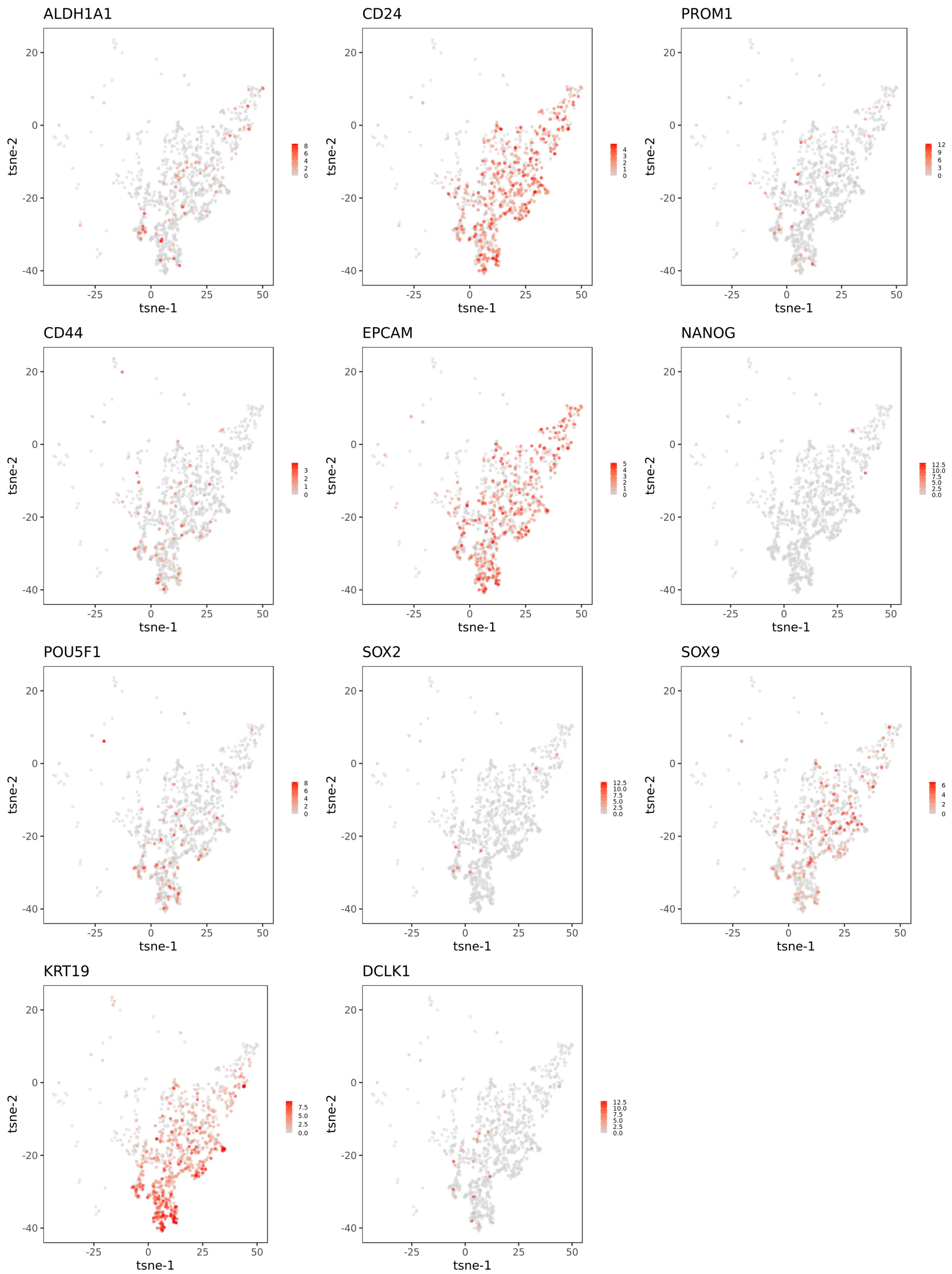

Supplemental Figure S2

S2C

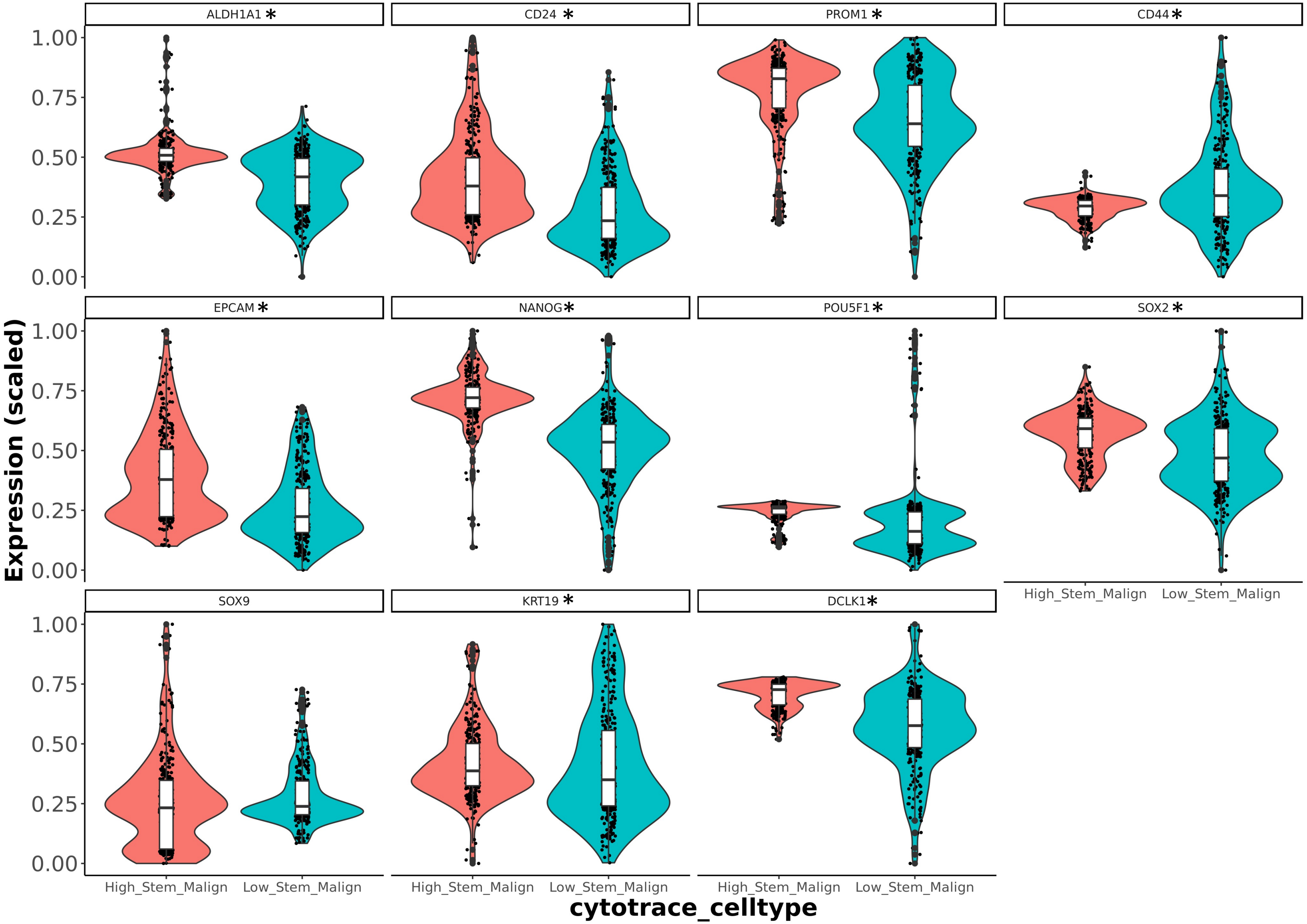

Supplemental Figure S3

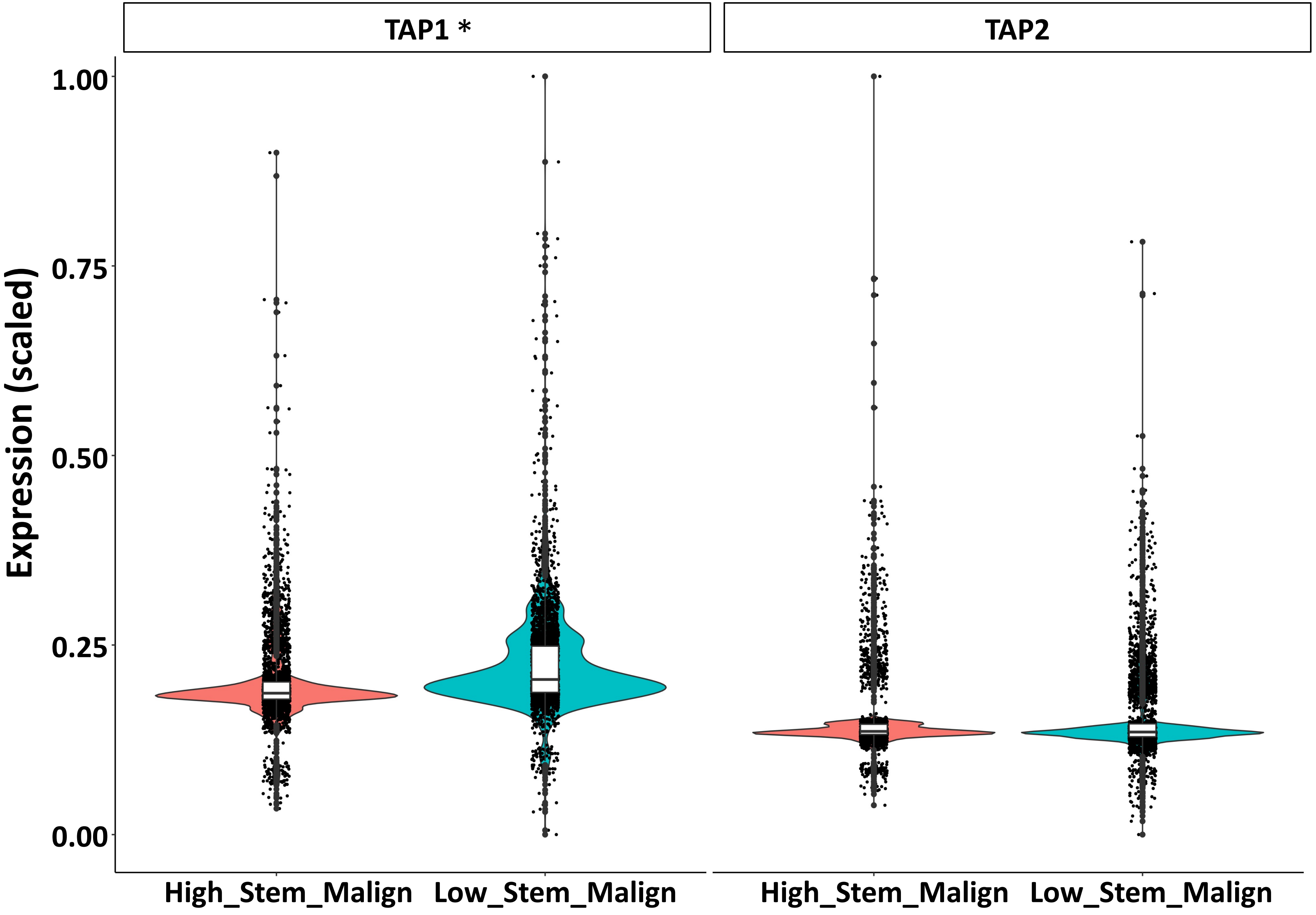

Supplemental Figure S4

S4A

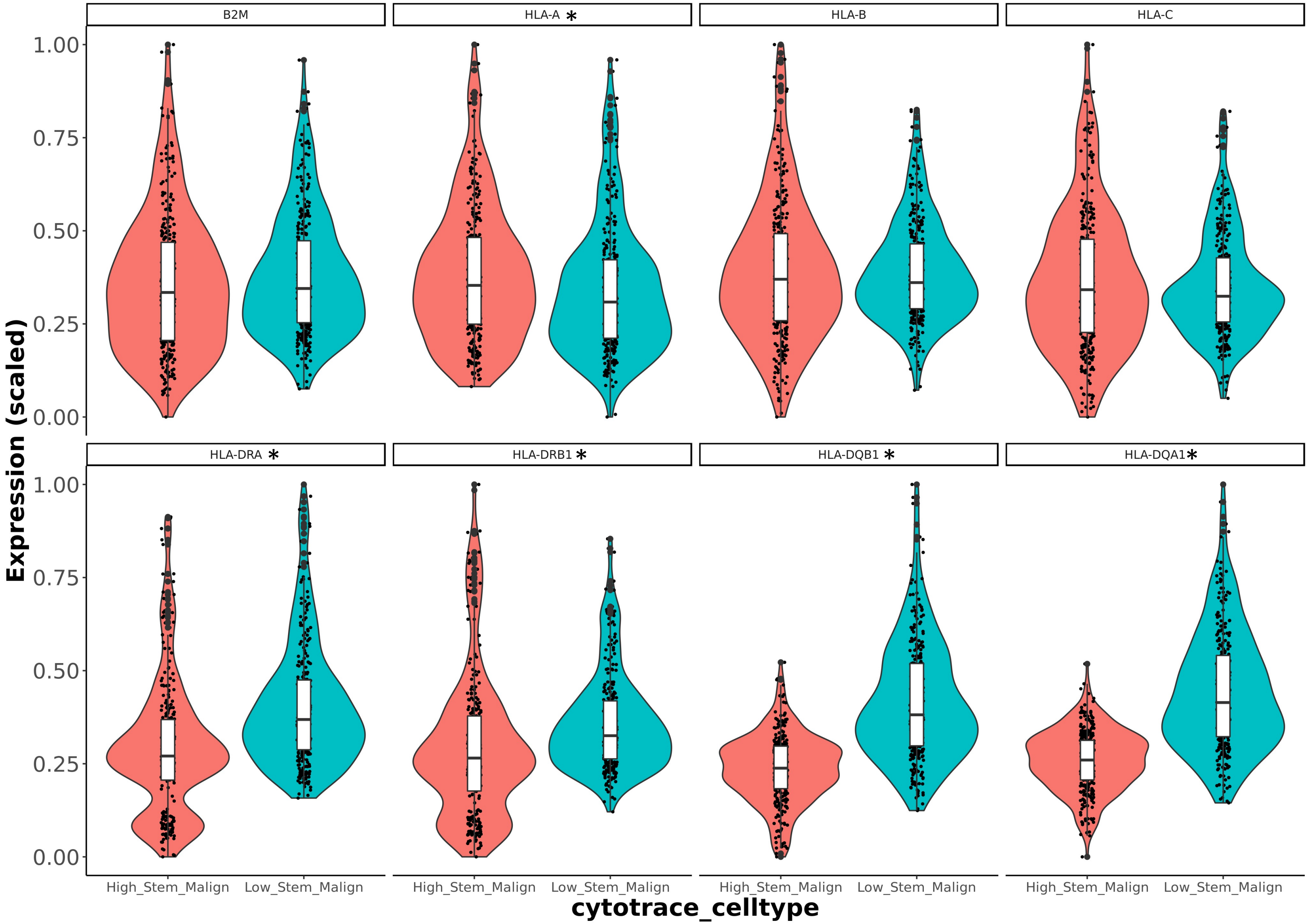

Supplemental Figure S4

S4B

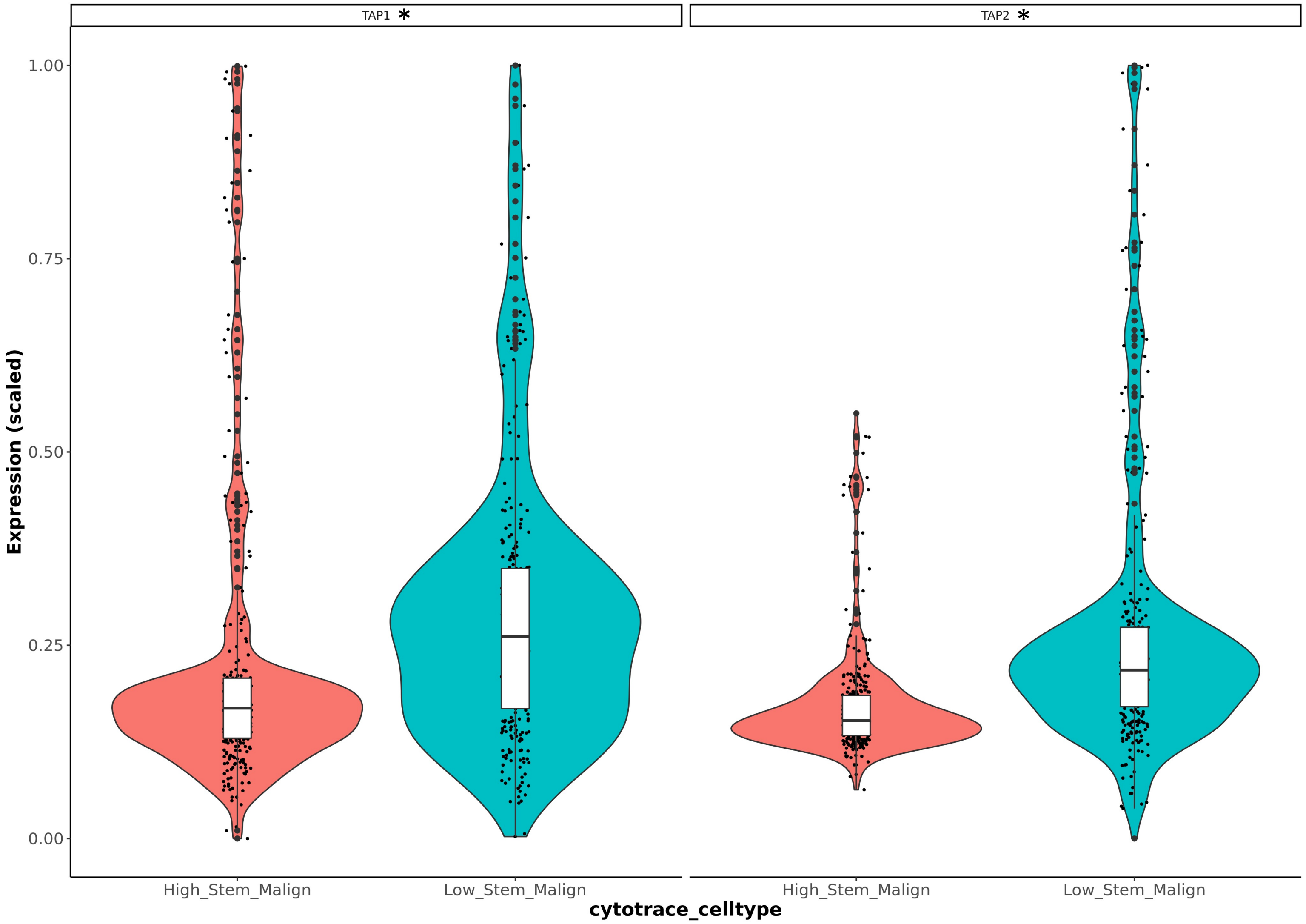

Supplemental Figure S5

S5A

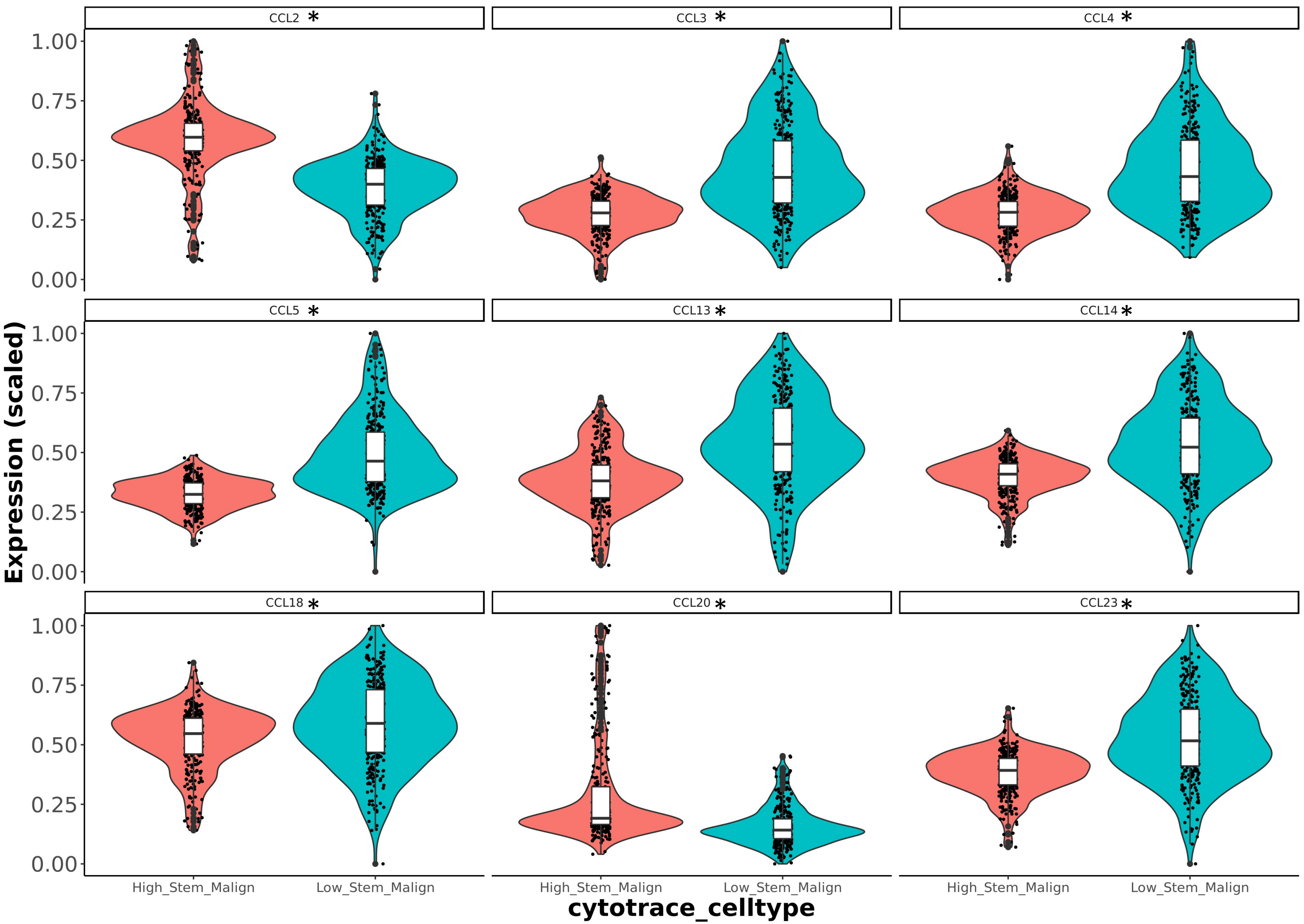

Supplemental Figure S5

S5B

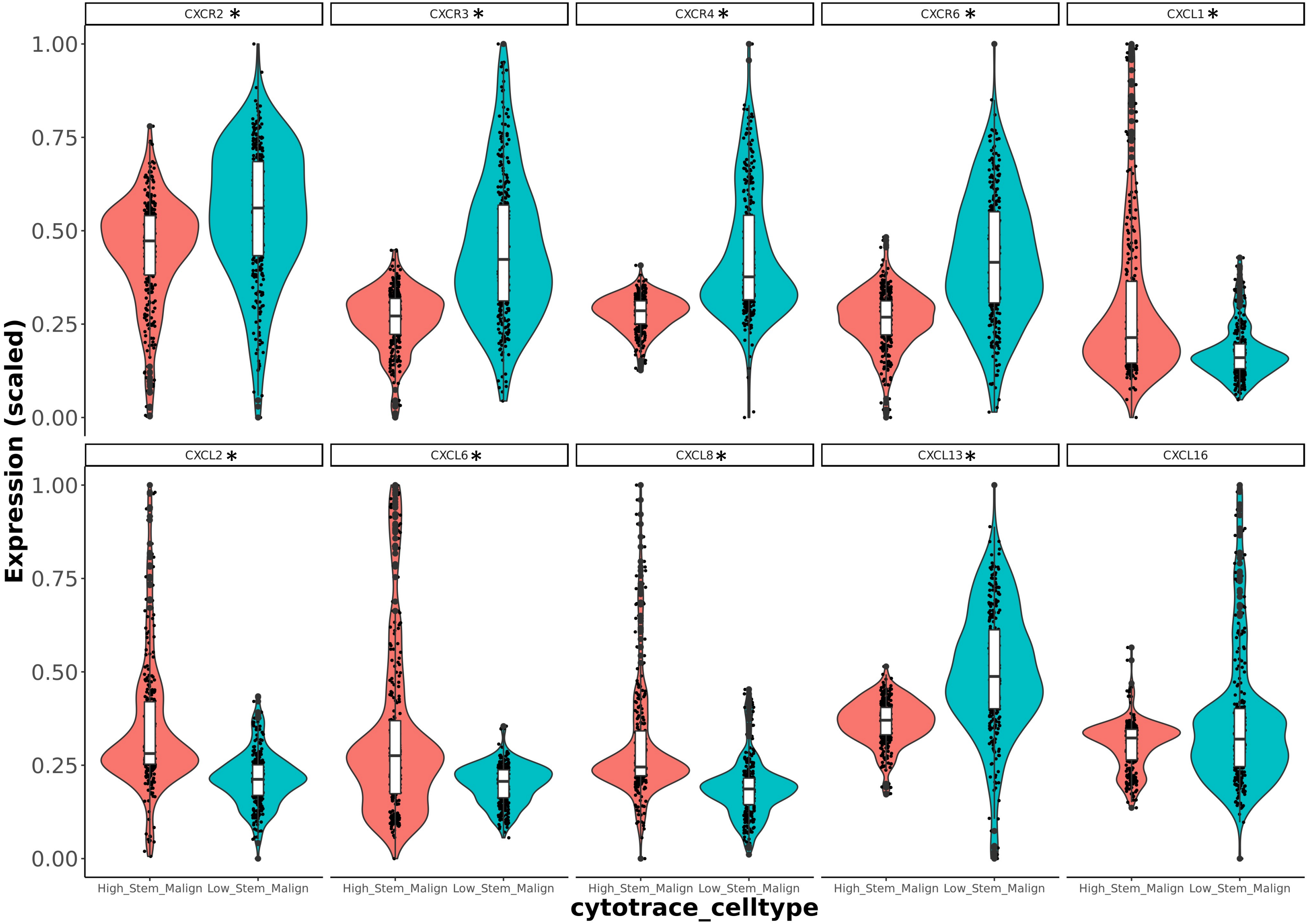

Supplemental Figure S5

S5C

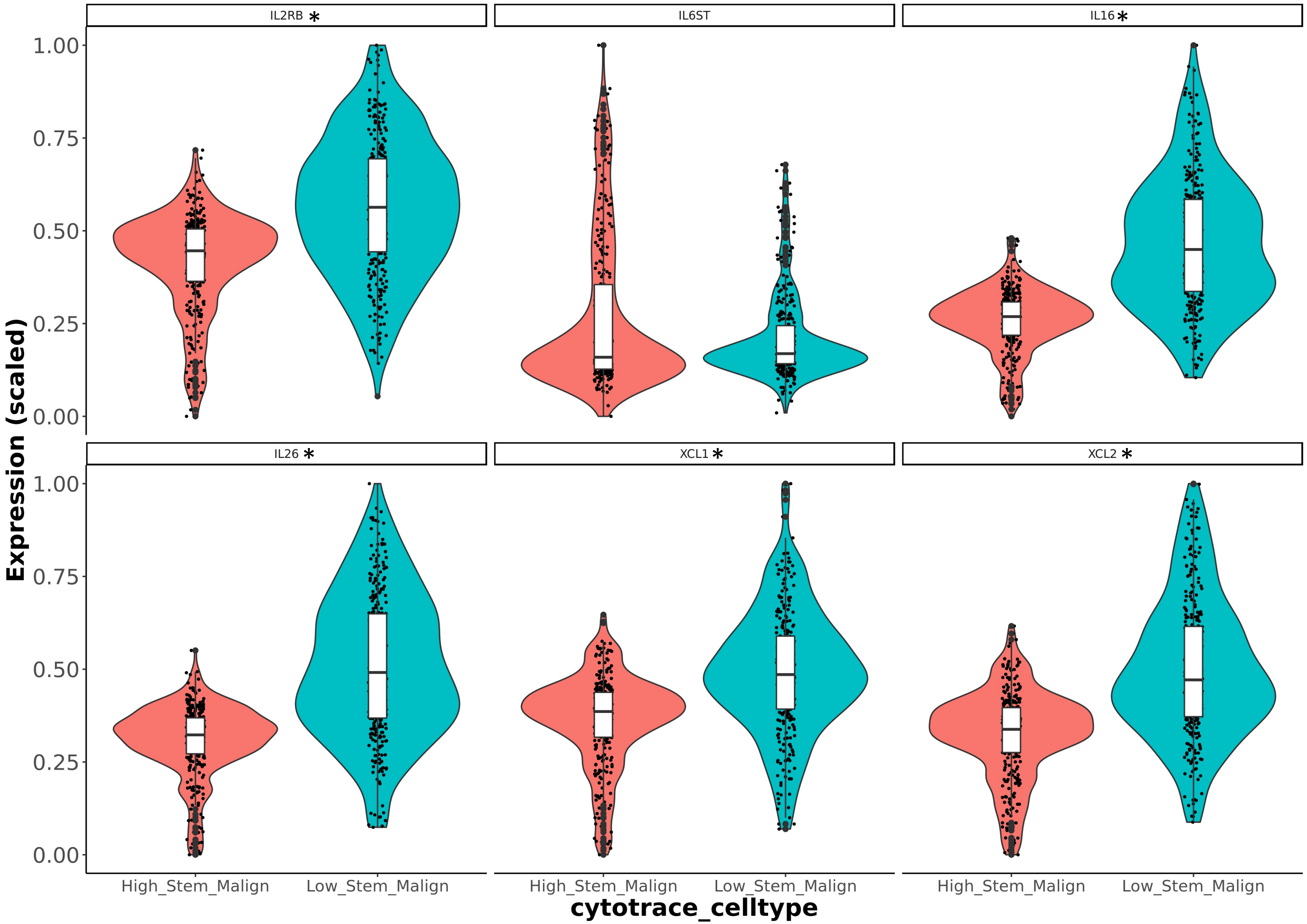

Supplemental Figure S5

S5D

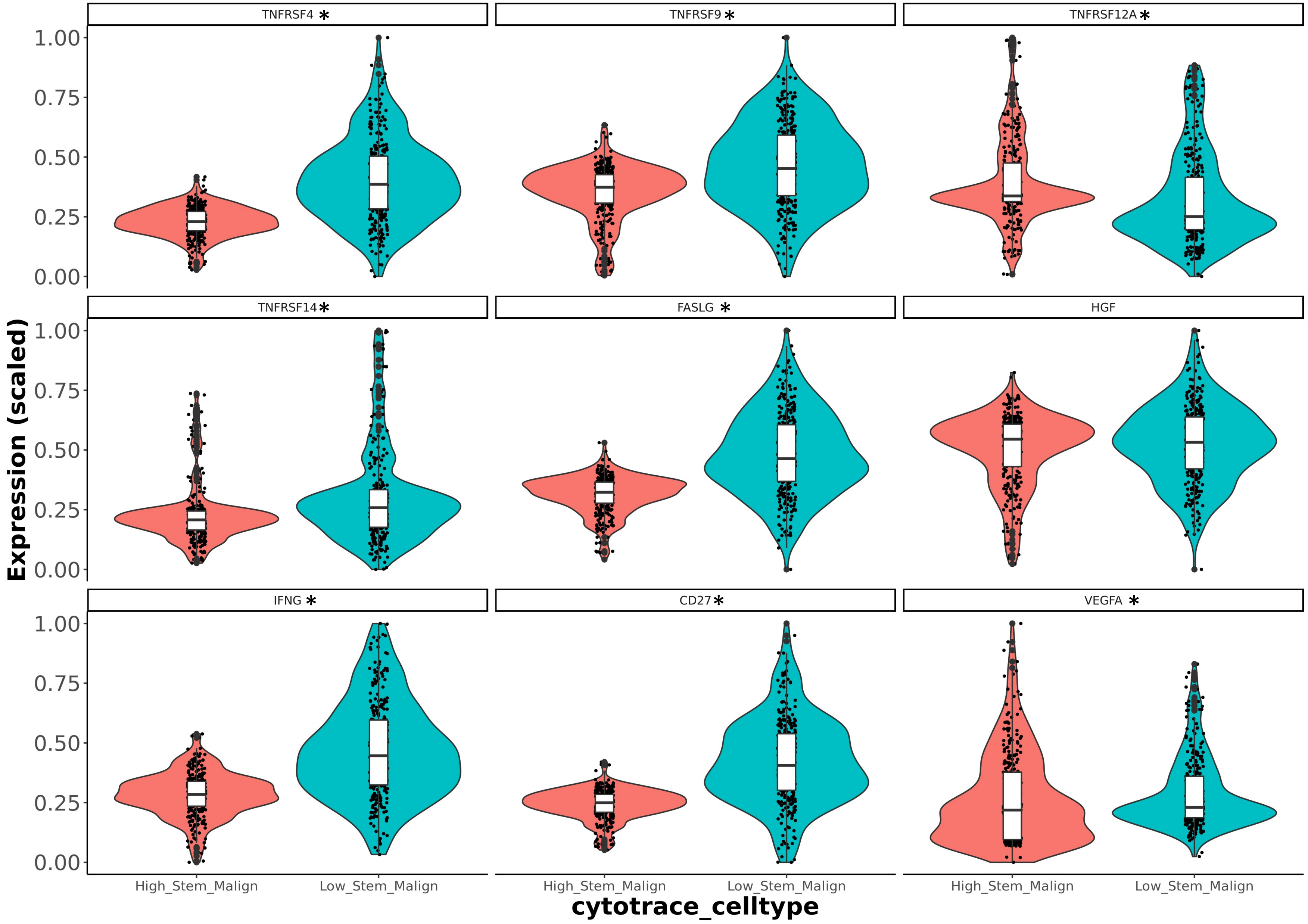

Supplement: Supplementary Materials — Supplemental Table S1: malignant cell counts from 5 tumor samples. Supplemental Table S2: collection of stemness signatures from the previous publications. Supplemental Table S3: fold change of genes between high stemness and low stemness malignant cells and P value from GSE138709. Supplemental Table S4: fold change of genes between high stemness and low stemness malignant cells and P value from GSE125449. Supplemental Figure S1: representative immunofluorescence images. Bar = 37.74 μm. Supplemental Figure S2: differentiation heterogeneity of malignant cells in iCCA from GSE125449. S2A: tSNE plots for malignant cells showing CytoTRACE analysis of malignant cells. S2B: tSNE plots showing the expression of CSC marker genes. S2C: violin plots showing the expression of CSC marker genes. ∗ indicates P < 0.05. Supplemental Figure S3: comparison of TAP1 and TAP2 between high stemness and low stemness iCCA cells from GSE138709, shown with violin plot. ∗ indicates P < 0.05. Supplemental Figure S4: comparison of MHC pathway profile between high stemness and low stemness iCCA cells from GSE125449. S4A: violin plot of MHC I and II pathway-related genes. S4B: violin plot of TAP1 and TAP2. ∗ indicates P < 0.05. Supplemental Figure S5: comparison of inflammatory factors between high stemness and low stemness iCCA cells from GSE125449. S5A: violin plot of C-C chemokines. S5B: violin plot of C-X-C chemokines. S5C: violin plot of interleukin family. S5D: TNF family and other inflammatory factors. ∗ indicates P < 0.05. [file 3558200.f1.zip › Supplemental Figures_031222.pdf]
