# Supplementary material for: Characterization of Immunogenicity of Malignant Cells with Stemness in Intrahepatic Cholangiocarcinoma by Single-Cell RNA Sequencing
Source: Stem Cells Int. 2022 Apr 29;2022:3558200. doi: 10.1155/2022/3558200 (PMC9076354; doi:10.1155/2022/3558200)
Supplement: Supplementary Materials — Supplemental Table S1: malignant cell counts from 5 tumor samples. Supplemental Table S2: collection of stemness signatures from the previous publications. Supplemental Table S3: fold change of genes between high stemness and low stemness malignant cells and P value from GSE138709. Supplemental Table S4: fold change of genes between high stemness and low stemness malignant cells and P value from GSE125449. Supplemental Figure S1: representative immunofluorescence images. Bar = 37.74 μm. Supplemental Figure S2: differentiation heterogeneity of malignant cells in iCCA from GSE125449. S2A: tSNE plots for malignant cells showing CytoTRACE analysis of malignant cells. S2B: tSNE plots showing the expression of CSC marker genes. S2C: violin plots showing the expression of CSC marker genes. ∗ indicates P < 0.05. Supplemental Figure S3: comparison of TAP1 and TAP2 between high stemness and low stemness iCCA cells from GSE138709, shown with violin plot. ∗ indicates P < 0.05. Supplemental Figure S4: comparison of MHC pathway profile between high stemness and low stemness iCCA cells from GSE125449. S4A: violin plot of MHC I and II pathway-related genes. S4B: violin plot of TAP1 and TAP2. ∗ indicates P < 0.05. Supplemental Figure S5: comparison of inflammatory factors between high stemness and low stemness iCCA cells from GSE125449. S5A: violin plot of C-C chemokines. S5B: violin plot of C-X-C chemokines. S5C: violin plot of interleukin family. S5D: TNF family and other inflammatory factors. ∗ indicates P < 0.05. [file 3558200.f1.zip › Supplemental tables (1).docx]

**Supplemental Table 1 Malignant cell count from individual tumor samples**

| **Tumor ID** | **Malignant cells** | **No. of top 25% high CytoTRACE score cells** | **No. of bottom 25% CytoTRACE score cells** |
| --- | --- | --- | --- |
| ICC18 | 5563 | 1601 | 1351 |
| ICC20 | 2273 | 543 | 328 |
| ICC23 | 891 | 171 | 270 |
| ICC24-1 | 619 | 152 | 226 |
| ICC24-2 | 2647 | 526 | 825 |
| **Total** | 11993 | 2993 | 3000 |

**Supplemental Table S2 Collection of stemness signatures from previous publications**

| **Palmer_Genome Bio_Cancer stemness[1]** | **Shats_Cancer Res_CSR[2]** | **Smith_Human Epithelial ASC[3]** | **Tathiane_Cell_stemness indice_CHOL[4]** | **Yan_PNAS_CD133GBM[5]** | **Miranda_PNAS_Cancer Stemness[6]** |
| --- | --- | --- | --- | --- | --- |
| ABCG1 | ASPM | ACSL4 | ACBD7 | AKAP4 | ACTRT3 |
| ACTRT3 | ATAD2 | ADCY3 | ADAMTS2 | ARHGAP11A | ADH5 |
| ADH5 | BIRC5 | AKR1B1 | AFF2 | ASPM | ALX1 |
| AFTPH | BUB1 | ARHGAP25 | AIFM3 | BARD1 | APLP1 |
| ALX1 | BUB1B | C20orf27 | ALDH1A3 | BIRC5 | ARMC9 |
| APLP1 | C10ORF26 | CACHD1 | ALPPL2 | BRCA1 | ARMCX2 |
| ARHGDIB | C21ORF45 | CCNB1IP1 | ANGPTL2 | C12orf32 | ASCC3 |
| ARMC9 | CCDC99 | CDCA7 | ANKRD50 | C17orf80 | ATP11C |
| ARMCX2 | CCNA2 | CDK6 | ANTXR1 | C2orf48 | BBS9 |
| ARRB2 | CCNB1 | DDX46 | AOC3 | C4orf21 | BCKDHB |
| ASCC3 | CCNB2 | DNMT1 | ARHGAP36 | CAPN14 | BMPR1A |
| ATP11C | CDC2 | DSE | ASCL2 | CASC5 | BOD1 |
| BBS9 | CDC20 | FAM216A | ASPN | CCDC102A | C14orf119 |
| BCAT1 | CDC7 | FBL | ASRGL1 | CCDC111 | C14orf166 |
| BCKDHB | CDKN3 | FCHSD2 | ATP1A3 | CCDC15 | CCL26 |
| BMPR1A | CENPA | FGD1 | ATP2A1 | CDCA2 | CDC123 |
| BOD1 | CENPF | GMPS | ATP4A | CDKN3 | CDH6 |
| BUB1B | CEP55 | GPX7 | AURKA | CENPH | CENPH |
| C10orf128 | CIRBP | HELLS | BGN | CENPK | CENPI |
| C14orf119 | CKS1B | IKBIP | BIRC5 | CKAP2L | CPSF3 |
| C14orf166 | CKS2 | ILF3 | BLM | CKS2 | DACT1 |
| C1orf135 | CST3 | JAM3 | C10orf10 | CTNNAL1 | DCUN1D5 |
| C4orf5 | DBF4 | KANK1 | C10orf99 | DHFR | DDX1 |
| C5orf56 | DCC1 | KDELC1 | C16orf59 | DHX57 | DHX15 |
| CCDC90B | DKFZP762E1312 | LGR6 | C17orf53 | DIAPH3 | DIAPH3 |
| CCDC99 | DLG7 | NAP1L1 | C1QL1 | DLGAP5 | DIMT1 |
| CCL26 | DTL | NASP | C21orf58 | DTL | DLGAP5 |
| CCNA2 | ECT2 | NKRF | C5orf34 | DTYMK | DNMT3B |
| CCNB1 | ESPL1 | NOL9 | CALB1 | ECT2 | DPH3 |
| CCNG1 | FBXO5 | NOTCH4 | CAMK2A | ENAH | DTD1 |
| CD53 | FEN1 | NUP107 | CCDC80 | FANCI | EIF2AK4 |
| CDC123 | FOXM1 | PFKM | CCL25 | FBXO5 | EIF2B3 |
| CDC25A | GPSM2 | PKD2 | CCNB2 | GGH | ERCC2 |
| CDH6 | GTPBP4 | PLTP | CCND3 | GINS2 | FAM118B |
| CENPE | HMGB2 | PRNP | CDCA8 | GMNN | FANCB |
| CENPH | HMMR | PTPN14 | CDH16 | H2AFZ | FGF2 |
| CENPI | IGFBP4 | PTTG1 | CELA2A | HMGB2 | FST |
| CHEK2 | IL6ST | RANBP1 | CENPA | IFNA17 | FZD2 |
| CMBL | ISG20L2 | RCC2 | CENPW | IFNA4 | GARS |
| CPSF3 | KIAA0101 | SAMD5 | CHODL | JAM2 | GBE1 |
| CTSH | KIAA1794 | SLC16A7 | CITED1 | KIAA0101 | GDF3 |
| CTSS | KIF11 | SLC41A1 | CKS2 | KIF11 | GNL2 |
| DACT1 | KIF14 | SLCO3A1 | CLCA1 | KIF15 | GPR176 |
| DBC1 | KIF15 | SUPT16H | CLEC3A | KIF2C | GPX8 |
| DCUN1D5 | KIF20A | TCOF1 | CLPS | KIF4A | HAS2 |
| DDX1 | KIF23 | TMEM201 | CNN1 | KNTC1 | HAT1 |
| DEPDC1B | KIF2C | TMEM237 | COL11A1 | LIG1 | HAUS1 |
| DHX15 | KIF4A | USP31 | COL1A2 | LMAN1L | HAUS6 |
| DIAPH3 | KNTC2 | VSNL1 | COL2A1 | LOC91431 | HDAC2 |
| DIMT1 | LAMB2 |  | COL3A1 | MAD2L1 | HDX |
| DLGAP5 | MAD2L1 |  | COL5A1 | MCM2 | HESX1 |
| DNMT3B | MCAM |  | COL5A2 | MCM3 | HMGA2 |
| DPH3 | MCM4 |  | COL6A3 | MELK | HTR7 |
| DTD1 | MCM6 |  | CPA1 | MND1 | IARS |
| EIF2AK4 | MELK |  | CPEB1 | MORN2 | ICMT |
| EIF2B3 | MLF1IP |  | CPZ | NACA | IGF2BP1 |
| ERCC2 | MSH2 |  | CRISPLD2 | NCAPH | INHBE |
| FAM118B | MTIF2 |  | CRYAB | NDC80 | IPO5 |
| FAM185BP | NCAPG |  | CSPG5 | NEK2 | KIF20A |
| FANCB | NCAPH |  | CST6 | NMU | KIF7 |
| FBXO22 | NEK2 |  | CTGF | NUF2 | LIN28B |
| FGF2 | NUSAP1 |  | CTRB1 | PBK | MED20 |
| FKSG49 | OIP5 |  | CTSE | PCNA | MIS18A |
| FST | PAICS |  | CUBN | POLQ | MMADHC |
| FYB | PBK |  | CYR61 | PRIM1 | MRPL3 |
| FZD2 | PDSS1 |  | DCN | PROM1 | MSH6 |
| GARS | PRC1 |  | DLC1 | PSG5 | MTHFD1L |
| GBE1 | PTTG1 |  | DLK1 | PTTG1 | MTHFD2 |
| GDF3 | PUS7 |  | DNAH11 | PTTG3 | MYCN |
| GIMAP1 | RACGAP1 |  | DPCR1 | PXMP2 | NMNAT2 |
| GIMAP5 | RAD51 |  | DPYSL3 | RAD51 | NREP |
| GIMAP6 | RAD51AP1 |  | E2F2 | RANBP1 | ORC1 |
| GIMAP7 | RCC1 |  | ECEL1 | RRM2 | OSTC |
| GIMAP8 | RFC4 |  | EFEMP2 | RTKN | PAICS |
| GNL2 | RRM2 |  | EFHD1 | SGOL1 | PDHB |
| GPR176 | SKP2 |  | EGR1 | SLC2A11 | PFAS |
| GPX8 | SMC2 |  | ELAVL3 | SMC2 | PLAA |
| HAS2 | SMC4 |  | ELN | SNRPE | PLRG1 |
| HAT1 | SNRPA1 |  | EME1 | SYTL4 | RAB3B |
| HAUS1 | SPTAN1 |  | EMILIN1 | TIMELESS | RAD1 |
| HAUS6 | STMN1 |  | EMP1 | TM4SF1 | RARS |
| HDAC2 | TALDO1 |  | EMX2OS | TMEM106C | RARS2 |
| HDX | TOP2A |  | ERCC6L | TOP2A | RIOK2 |
| HESX1 | TPX2 |  | F2R | TPX2 | ROR1 |
| HIST1H4C | TRIP13 |  | FAM101A | TRIP13 | RPF2 |
| HMGA2 | TTC13 |  | FAM72B | TROAP | RRAS2 |
| HTR7 | TTK |  | FAM72D | TTK | SAMM50 |
| IARS | UBE2C |  | FANCA | TYMS | SEC11A |
| ICMT | UCK2 |  | FBN1 | WDR34 | SKIV2L2 |
| IGF2BP1 | WDR67 |  | FN1 | SKIV2L2 | SLC24A1 |
| INHBE | ZWINT |  | FNDC1 | SLC24A1 | SLC25A3 |
| IPO5 |  |  | FOSB | SLC25A3 | SNRPD3 |
| JUNB |  |  | FOSL1 | SNRPD3 | SNX8 |
| KDELC1 |  |  | FOXL1 | SNX8 | SOHLH2 |
| KIAA0020 |  |  | FSTL1 | SOHLH2 | TBC1D16 |
| KIF11 |  |  | FUT9 | TBC1D16 | TBCE |
| KIF20A |  |  | FZD10 | TBCE | TET1 |
| KIF23 |  |  | GABRA3 | TET1 | TEX10 |
| KIF7 |  |  | GALNT5 | TEX10 | TM2D2 |
| LIN28B |  |  | GEM | TM2D2 | TRIM24 |
| LOC285431 |  |  | GLT8D2 | TRIM24 | TRNT1 |
| LOC400931 |  |  | GPR87 | TRNT1 | TRPC4 |
| MALAT1 |  |  | GUCY2C | TRPC4 | UBE2K |
| MCM10 |  |  | HEPH | UBE2K | UGGT2 |
| MCM4 |  |  | HJURP | UGGT2 | UTP6 |
| MCM6 |  |  | HMGA2 | UTP6 | XPOT |
| MED2 |  |  | HOXA7 | XPOT | XRCC5 |
| MIS18A |  |  | INHBA | XRCC5 | XXYLT1 |
| MLLT6 |  |  | ITGBL1 | XXYLT1 | ZNF788 |
| MMADHC |  |  | KCNC2 | ZNF788 |  |
| MRPL3 |  |  | KCNE4 |  |  |
| MS4A7 |  |  | KIAA0101 |  |  |
| MSH2 |  |  | KIAA1524 |  |  |
| MSH6 |  |  | KIF18B |  |  |
| MTHFD1L |  |  | KLK2 |  |  |
| MTHFD2 |  |  | KRT20 |  |  |
| MXI1 |  |  | KRT75 |  |  |
| MYCN |  |  | L1CAM |  |  |
| NA |  |  | LAMA2 |  |  |
| NDC80 |  |  | LIX1 |  |  |
| NMNAT2 |  |  | LRRC32 |  |  |
| NPR3 |  |  | LRRN1 |  |  |
| NREP |  |  | LUM |  |  |
| NUSAP1 |  |  | MAMDC2 |  |  |
| ORC1 |  |  | ME1 |  |  |
| OSTC |  |  | MEP1A |  |  |
| PAICS |  |  | MGAT5B |  |  |
| PDE4C |  |  | MGP |  |  |
| PDHB |  |  | MIOX |  |  |
| PECAM1 |  |  | MMP2 |  |  |
| PFAS |  |  | MMP28 |  |  |
| PILRB |  |  | MSI1 |  |  |
| PLAA |  |  | MUC13 |  |  |
| PLRG1 |  |  | MXRA5 |  |  |
| POLE2 |  |  | MXRA8 |  |  |
| PRIM1 |  |  | MYADM |  |  |
| PTPRC |  |  | MYB |  |  |
| RAB3B |  |  | MYH11 |  |  |
| RAD1 |  |  | MYO1A |  |  |
| RARS |  |  | MYOM3 |  |  |
| RARS2 |  |  | NAPSA |  |  |
| RCSD1 |  |  | NCAM1 |  |  |
| RGCC |  |  | NEFM |  |  |
| RIOK2 |  |  | NPY |  |  |
| ROR1 |  |  | NUSAP1 |  |  |
| RPF2 |  |  | ODAM |  |  |
| RRAS2 |  |  | OIP5 |  |  |
| RSAD2 |  |  | PCYT1B |  |  |
| SAMM50 |  |  | PDE1A |  |  |
| SAT1 |  |  | PDLIM3 |  |  |
| SEC11A |  |  | PGAM2 |  |  |
| SKIV2L2 |  |  | PGC |  |  |
| SKP2 |  |  | PIF1 |  |  |
| SLC24A1 |  |  | PLAT |  |  |
| SLC25A3 |  |  | PLCXD2 |  |  |
| SMARCC2 |  |  | PNLIP |  |  |
| SNRPD3 |  |  | PODN |  |  |
| SNX8 |  |  | POLQ |  |  |
| SOHLH2 |  |  | POSTN |  |  |
| TARDBP |  |  | PPAPDC1A |  |  |
| TBC1D16 |  |  | PPY |  |  |
| TBCE |  |  | PRKG1 |  |  |
| TET1 |  |  | PRSS1 |  |  |
| TEX10 |  |  | PSD2 |  |  |
| TM2D2 |  |  | PTTG1 |  |  |
| TRIM24 |  |  | PTX3 |  |  |
| TRNT1 |  |  | RCN3 |  |  |
| TRPC4 |  |  | RNF212 |  |  |
| TSHZ2 |  |  | ROR2 |  |  |
| UBE2K |  |  | RRM2 |  |  |
| UBXN2A |  |  | S100A9 |  |  |
| UGGT2 |  |  | SCGB1D2 |  |  |
| UTP6 |  |  | SCUBE3 |  |  |
| WASH3P |  |  | SEMA3D |  |  |
| XPOT |  |  | SERPINB2 |  |  |
| XRCC5 |  |  | SGOL1 |  |  |
| XXYLT1 |  |  | SHOX2 |  |  |
| ZNF167 |  |  | SKA1 |  |  |
| ZNF788 |  |  | SKA3 |  |  |
|  |  |  | SLC26A3 |  |  |
|  |  |  | SLC26A7 |  |  |
|  |  |  | SLC5A10 |  |  |
|  |  |  | SLC9A3 |  |  |
|  |  |  | SNHG3 |  |  |
|  |  |  | SP6 |  |  |
|  |  |  | SPARC |  |  |
|  |  |  | SSC5D |  |  |
|  |  |  | STMN2 |  |  |
|  |  |  | STON1 |  |  |
|  |  |  | SULT1C2 |  |  |
|  |  |  | TAC3 |  |  |
|  |  |  | TACSTD2 |  |  |
|  |  |  | TCAP |  |  |
|  |  |  | TGFB1I1 |  |  |
|  |  |  | THBS1 |  |  |
|  |  |  | THBS2 |  |  |
|  |  |  | TIMP3 |  |  |
|  |  |  | TK1 |  |  |
|  |  |  | TM4SF20 |  |  |
|  |  |  | TMEM151B |  |  |
|  |  |  | TRIM9 |  |  |
|  |  |  | TRIP13 |  |  |
|  |  |  | TTK |  |  |
|  |  |  | VCAN |  |  |
|  |  |  | VSIG2 |  |  |
|  |  |  | WBSCR17 |  |  |
|  |  |  | WNT7A |  |  |
|  |  |  | ZDHHC11 |  |  |
|  |  |  | ZNF296 |  |  |

**Reference:**

1 Palmer NP, Schmid PR, Berger B and Kohane IS. A gene expression profile of stem cell pluripotentiality and differentiation is conserved across diverse solid and hematopoietic cancers. Genome biology 2012;13:R71-R71. PMID:22909066. doi: 10.1186/gb-2012-13-8-r71.

2 Shats I, Gatza ML, Chang JT, Mori S, Wang J, Rich J, et al. Using a Stem Cell–Based Signature to Guide Therapeutic Selection in Cancer. Cancer Research 2011;71:1772-1780. doi: 10.1158/0008-5472.Can-10-1735.

3 Smith BA, Balanis NG, Nanjundiah A, Sheu KM, Tsai BL, Zhang Q, et al. A Human Adult Stem Cell Signature Marks Aggressive Variants across Epithelial Cancers. Cell Reports 2018;24:3353-3366.e5. doi: <https://doi.org/10.1016/j.celrep.2018.08.062>.

4 Malta TM, Sokolov A, Gentles AJ, Burzykowski T, Poisson L, Weinstein JN, et al. Machine Learning Identifies Stemness Features Associated with Oncogenic Dedifferentiation. Cell 2018;173:338-354.e15. doi: 10.1016/j.cell.2018.03.034.

5 Yan X, Ma L, Yi D, Yoon J-g, Diercks A, Foltz G, et al. A CD133-related gene expression signature identifies an aggressive glioblastoma subtype with excessive mutations. Proceedings of the National Academy of Sciences 2011;108:1591-1596. doi: 10.1073/pnas.1018696108.

6 Miranda A, Hamilton PT, Zhang AW, Pattnaik S, Becht E, Mezheyeuski A, et al. Cancer stemness, intratumoral heterogeneity, and immune response across cancers. Proceedings of the National Academy of Sciences 2019;116:9020-9029. doi: 10.1073/pnas.1818210116.

**Supplemental Table S3 Fold change of genes between high stemness and low stemness malignant cells and p value from GSE138709**

|  | gene | p_adj_val | high_stem.low_stem.mean_diff |
| --- | --- | --- | --- |
| 1 | TAP1 | 2.50E-115 | -0.253803693 |
| 2 | TAP2 | 0.05970355 | -0.06327024 |
| 3 | B2M | 7.84E-257 | -1.001437313 |
| 4 | HLA-A | 0 | -0.923203791 |
| 5 | HLA-B | 0 | -0.943526133 |
| 6 | HLA-C | 0 | -0.77803203 |
| 7 | HLA-DRA | 0.18197405 | -0.341945064 |
| 8 | HLA-DRB1 | 1.88E-39 | -0.501404624 |
| 9 | HLA-DQB1 | 3.09E-28 | -0.316800302 |
| 10 | HLA-DQA1 | 1.47E-40 | -0.306746358 |
| 11 | CCL2 | 1.05E-175 | 0.112196053 |
| 12 | CCL3 | 1.41E-120 | -0.384493561 |
| 13 | CCL4 | 0 | -1.518124997 |
| 14 | CCL5 | 0 | -3.56666213 |
| 15 | CCL7 | 1.52E-228 | -0.064893614 |
| 16 | CCL13 | 1.51E-70 | -0.001043468 |
| 17 | CCL14 | 1.89E-85 | -0.23340214 |
| 18 | CCL18 | 9.67E-62 | 0.014978845 |
| 19 | CCL20 | 1.18E-07 | 0.028729374 |
| 20 | CCL23 | 1.38E-240 | -0.064987826 |
| 21 | CXCR2 | 0.19610678 | 0.02613741 |
| 22 | CXCR3 | 0 | -0.475062886 |
| 23 | CXCR4 | 0 | -3.047767207 |
| 24 | CXCR6 | 0 | -0.779057245 |
| 25 | CXCL1 | 8.25E-132 | 1.348505235 |
| 26 | CXCL2 | 2.76E-128 | 0.978514963 |
| 27 | CXCL6 | 5.41E-149 | 0.576073526 |
| 28 | CXCL8 | 1.85E-67 | 2.161919462 |
| 29 | CXCL13 | 0 | -0.402392738 |
| 30 | CXCL16 | 3.31E-11 | 0.123172089 |
| 31 | IL2RB | 0 | -0.645326341 |
| 32 | IL6ST | 3.32E-05 | 0.035006127 |
| 33 | IL13 | 7.88E-46 | -0.006893579 |
| 34 | IL16 | 0 | -0.364953776 |
| 35 | IL21 | 2.07E-150 | -0.028352477 |
| 36 | IL22 | 0.00143302 | 0.000668195 |
| 37 | IL26 | 1.18E-268 | -0.098131436 |
| 38 | XCL1 | 0 | -0.543676829 |
| 39 | XCL2 | 0 | -0.982185187 |
| 40 | TNFRSF4 | 0 | -0.309187618 |
| 41 | TNFRSF6B | 5.21E-14 | 0.096708853 |
| 42 | TNFRSF9 | 0 | -0.617887967 |
| 43 | TNFRSF12A | 6.58E-52 | 0.77087645 |
| 44 | TNFRSF14 | 6.77E-29 | -0.093678509 |
| 45 | FASLG | 0 | -0.506473072 |
| 46 | HGF | 7.69E-139 | 0.485855776 |
| 47 | IFNG | 0 | -0.766835512 |
| 48 | CD27 | 1.81E-259 | -0.386333177 |
| 49 | VEGFA | 2.51E-58 | 0.272268054 |
| 50 | ALDH1A1 | 1.32E-296 | 0.408333414 |
| 51 | CD24 | 6.36E-128 | 1.104442096 |
| 52 | PROM1 | 5.87E-103 | -0.040931446 |
| 53 | CD44 | 5.07E-128 | -0.52655275 |
| 54 | EPCAM | 3.43E-129 | 0.992788912 |
| 55 | SALL4 | 2.44E-74 | -0.124908426 |
| 56 | NANOG | 6.20E-48 | -0.033216915 |
| 57 | POU5F1 | 2.10E-112 | 0.054987287 |
| 58 | SOX2 | 1.23E-52 | 0.07358976 |
| 59 | SOX9 | 6.71E-61 | 0.493631227 |
| 60 | KRT19 | 0 | 5.733031767 |
| 61 | DCLK1 | 5.80E-188 | -0.023303489 |

**Supplemental Table S4 Fold change of genes between high stemness and low stemness malignant cells and p value from GSE125449**

|  | gene | p_adj_val | high_stem.low_stem.mean_diff |
| --- | --- | --- | --- |
| 1 | TAP1 | 7.75E-10 | -0.137813589 |
| 2 | TAP2 | 9.04E-19 | -0.348699459 |
| 3 | B2M | 0.09976994 | -0.071708019 |
| 4 | HLA-A | 0.01070923 | 0.0968085 |
| 5 | HLA-B | 0.43981769 | 0.003158998 |
| 6 | HLA-C | 0.89748417 | -0.006072395 |
| 7 | HLA-DRA | 3.63E-14 | -0.462842396 |
| 8 | HLA-DRB1 | 5.69E-10 | -0.231826661 |
| 9 | HLA-DQB1 | 8.91E-40 | -0.250034919 |
| 10 | HLA-DQA1 | 4.46E-45 | -0.315751266 |
| 11 | CCL2 | 4.64E-37 | 0.536804764 |
| 12 | CCL3 | 5.09E-35 | -0.24719831 |
| 13 | CCL4 | 8.02E-35 | -0.267407821 |
| 14 | CCL5 | 4.46E-45 | -0.379060485 |
| 15 | CCL13 | 1.10E-27 | -0.085926673 |
| 16 | CCL14 | 6.35E-23 | -0.079351772 |
| 17 | CCL18 | 8.86E-07 | -0.043954162 |
| 18 | CCL20 | 2.02E-22 | 2.028511992 |
| 19 | CCL23 | 1.41E-26 | -0.023310403 |
| 20 | CXCR2 | 2.89E-12 | -0.00633763 |
| 21 | CXCR3 | 2.77E-41 | -0.174721624 |
| 22 | CXCR4 | 2.40E-45 | -0.543575285 |
| 23 | CXCR6 | 9.34E-40 | -0.0813642 |
| 24 | CXCL1 | 2.86E-15 | 1.499823316 |
| 25 | CXCL2 | 6.11E-39 | 1.872154074 |
| 26 | CXCL6 | 8.47E-13 | 1.741693482 |
| 27 | CXCL8 | 2.03E-34 | 1.921279503 |
| 28 | CXCL13 | 7.13E-33 | -0.16389547 |
| 29 | CXCL16 | 0.07818408 | -0.204012168 |
| 30 | IL2RB | 8.21E-23 | -0.107378413 |
| 31 | IL6ST | 0.37467107 | 0.111380417 |
| 32 | IL16 | 8.92E-50 | -0.198236675 |
| 33 | IL26 | 2.52E-43 | -0.029307157 |
| 34 | XCL1 | 5.09E-26 | -0.089893317 |
| 35 | XCL2 | 1.11E-32 | -0.088850349 |
| 36 | TNFRSF4 | 1.58E-43 | -0.325123186 |
| 37 | TNFRSF9 | 1.43E-14 | -0.061528324 |
| 38 | TNFRSF12A | 1.70E-12 | 0.885040478 |
| 39 | TNFRSF14 | 8.49E-06 | -0.128156023 |
| 40 | FASLG | 4.17E-43 | -0.067863704 |
| 41 | HGF | 0.26043304 | -0.024261213 |
| 42 | IFNG | 1.59E-35 | -0.173260654 |
| 43 | CD27 | 1.85E-46 | -0.377177166 |
| 44 | VEGFA | 0.00088175 | -0.200386441 |
| 45 | ALDH1A1 | 7.25E-22 | 0.719577715 |
| 46 | CD24 | 5.03E-20 | 1.222496807 |
| 47 | PROM1 | 1.85E-11 | 0.039309073 |
| 48 | CD44 | 1.78E-10 | -0.492042127 |
| 49 | EPCAM | 4.14E-17 | 1.17288104 |
| 50 | NANOG | 4.41E-30 | 0.129205656 |
| 51 | POU5F1 | 4.16E-18 | -0.157046827 |
| 52 | SOX2 | 1.78E-10 | 0.066041887 |
| 53 | SOX9 | 0.1576796 | -0.090830765 |
| 54 | KRT19 | 0.00379844 | 0.388741398 |
| 55 | DCLK1 | 3.66E-22 | -0.19807316 |
