# Supplementary figures and images for: Characterization of Immunogenicity of Malignant Cells with Stemness in Intrahepatic Cholangiocarcinoma by Single-Cell RNA Sequencing
Source: Stem Cells Int. 2022 Apr 29;2022:3558200. doi: 10.1155/2022/3558200 (PMC9076354; doi:10.1155/2022/3558200)

**A**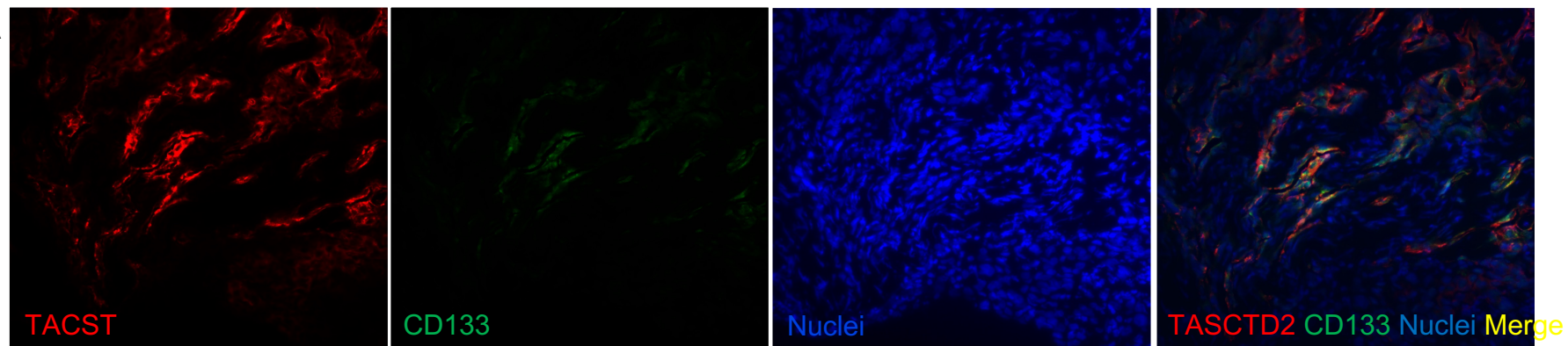**B**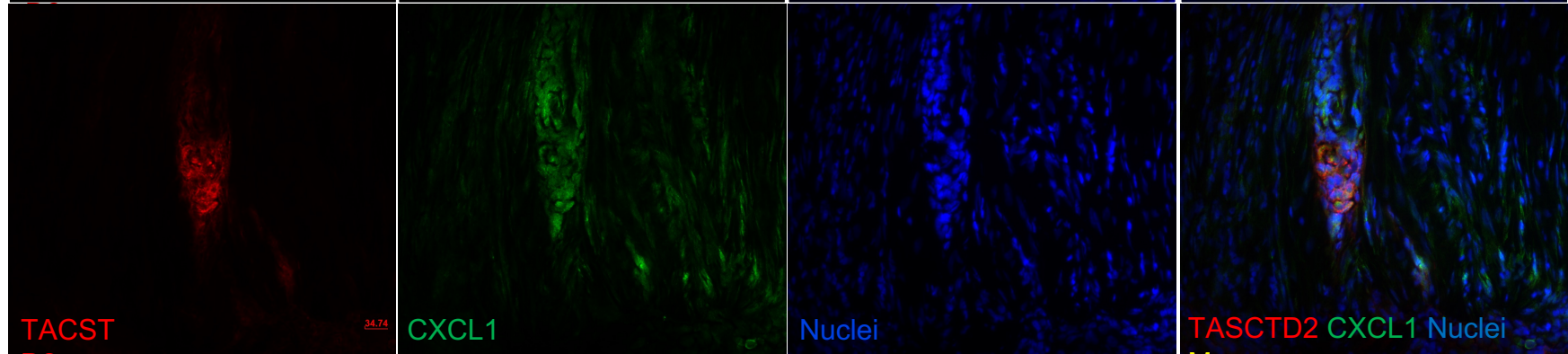**C**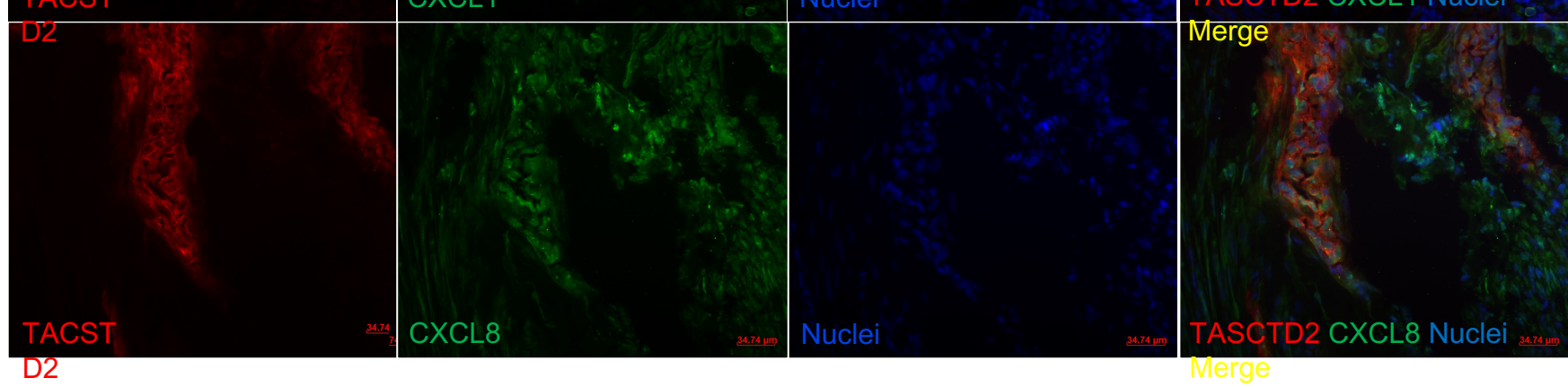

Supplement: Supplementary Materials — Supplemental Table S1: malignant cell counts from 5 tumor samples. Supplemental Table S2: collection of stemness signatures from the previous publications. Supplemental Table S3: fold change of genes between high stemness and low stemness malignant cells and P value from GSE138709. Supplemental Table S4: fold change of genes between high stemness and low stemness malignant cells and P value from GSE125449. Supplemental Figure S1: representative immunofluorescence images. Bar = 37.74 μm. Supplemental Figure S2: differentiation heterogeneity of malignant cells in iCCA from GSE125449. S2A: tSNE plots for malignant cells showing CytoTRACE analysis of malignant cells. S2B: tSNE plots showing the expression of CSC marker genes. S2C: violin plots showing the expression of CSC marker genes. ∗ indicates P < 0.05. Supplemental Figure S3: comparison of TAP1 and TAP2 between high stemness and low stemness iCCA cells from GSE138709, shown with violin plot. ∗ indicates P < 0.05. Supplemental Figure S4: comparison of MHC pathway profile between high stemness and low stemness iCCA cells from GSE125449. S4A: violin plot of MHC I and II pathway-related genes. S4B: violin plot of TAP1 and TAP2. ∗ indicates P < 0.05. Supplemental Figure S5: comparison of inflammatory factors between high stemness and low stemness iCCA cells from GSE125449. S5A: violin plot of C-C chemokines. S5B: violin plot of C-X-C chemokines. S5C: violin plot of interleukin family. S5D: TNF family and other inflammatory factors. ∗ indicates P < 0.05. [file 3558200.f1.zip › Supplemental figure S1.pdf]

**S2A**

cytotrace\_celltype

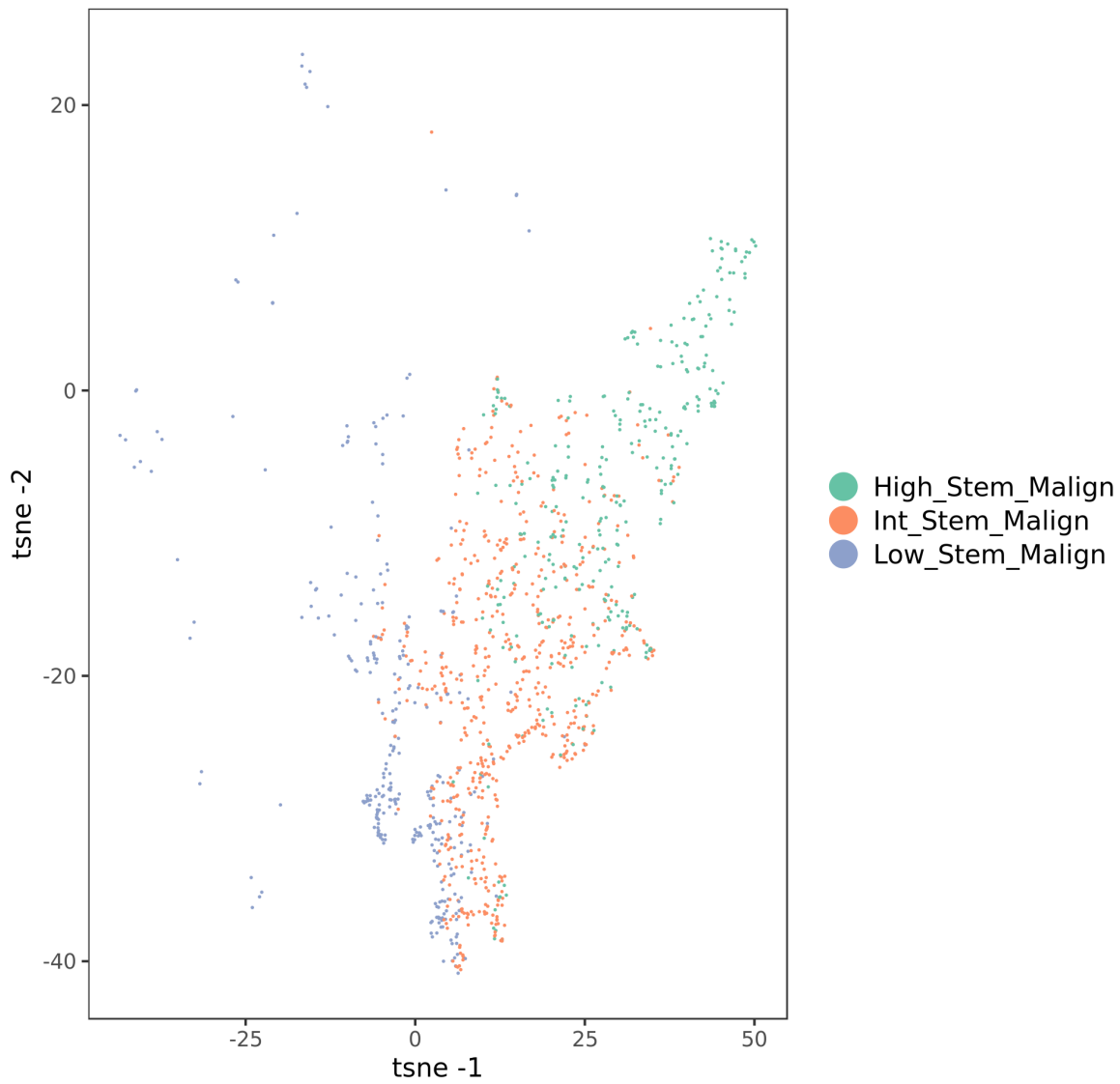

Supplement: Supplementary Materials — Supplemental Table S1: malignant cell counts from 5 tumor samples. Supplemental Table S2: collection of stemness signatures from the previous publications. Supplemental Table S3: fold change of genes between high stemness and low stemness malignant cells and P value from GSE138709. Supplemental Table S4: fold change of genes between high stemness and low stemness malignant cells and P value from GSE125449. Supplemental Figure S1: representative immunofluorescence images. Bar = 37.74 μm. Supplemental Figure S2: differentiation heterogeneity of malignant cells in iCCA from GSE125449. S2A: tSNE plots for malignant cells showing CytoTRACE analysis of malignant cells. S2B: tSNE plots showing the expression of CSC marker genes. S2C: violin plots showing the expression of CSC marker genes. ∗ indicates P < 0.05. Supplemental Figure S3: comparison of TAP1 and TAP2 between high stemness and low stemness iCCA cells from GSE138709, shown with violin plot. ∗ indicates P < 0.05. Supplemental Figure S4: comparison of MHC pathway profile between high stemness and low stemness iCCA cells from GSE125449. S4A: violin plot of MHC I and II pathway-related genes. S4B: violin plot of TAP1 and TAP2. ∗ indicates P < 0.05. Supplemental Figure S5: comparison of inflammatory factors between high stemness and low stemness iCCA cells from GSE125449. S5A: violin plot of C-C chemokines. S5B: violin plot of C-X-C chemokines. S5C: violin plot of interleukin family. S5D: TNF family and other inflammatory factors. ∗ indicates P < 0.05. [file 3558200.f1.zip › Supplemental figure S2A.pdf]

# S2B

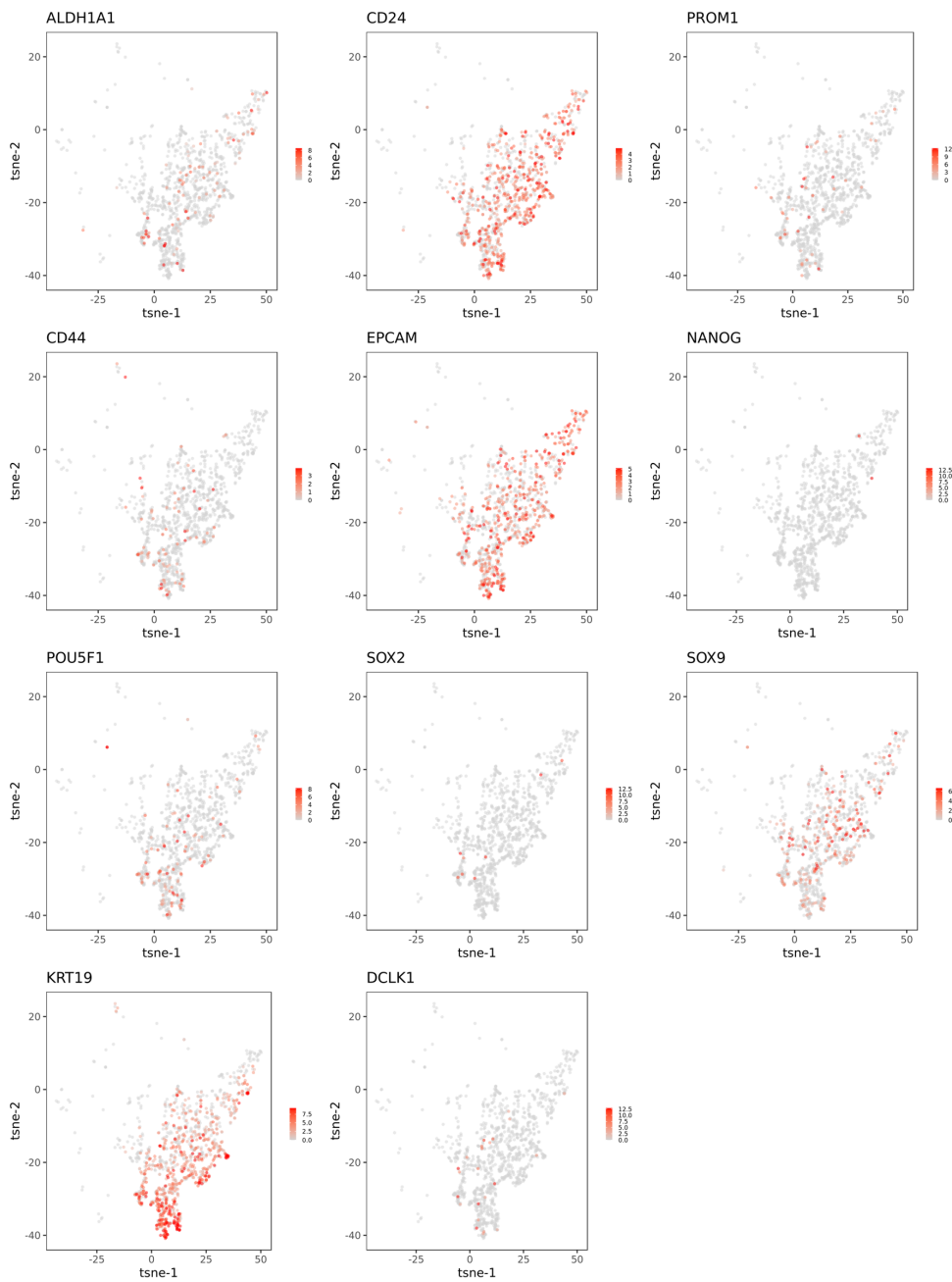

Supplement: Supplementary Materials — Supplemental Table S1: malignant cell counts from 5 tumor samples. Supplemental Table S2: collection of stemness signatures from the previous publications. Supplemental Table S3: fold change of genes between high stemness and low stemness malignant cells and P value from GSE138709. Supplemental Table S4: fold change of genes between high stemness and low stemness malignant cells and P value from GSE125449. Supplemental Figure S1: representative immunofluorescence images. Bar = 37.74 μm. Supplemental Figure S2: differentiation heterogeneity of malignant cells in iCCA from GSE125449. S2A: tSNE plots for malignant cells showing CytoTRACE analysis of malignant cells. S2B: tSNE plots showing the expression of CSC marker genes. S2C: violin plots showing the expression of CSC marker genes. ∗ indicates P < 0.05. Supplemental Figure S3: comparison of TAP1 and TAP2 between high stemness and low stemness iCCA cells from GSE138709, shown with violin plot. ∗ indicates P < 0.05. Supplemental Figure S4: comparison of MHC pathway profile between high stemness and low stemness iCCA cells from GSE125449. S4A: violin plot of MHC I and II pathway-related genes. S4B: violin plot of TAP1 and TAP2. ∗ indicates P < 0.05. Supplemental Figure S5: comparison of inflammatory factors between high stemness and low stemness iCCA cells from GSE125449. S5A: violin plot of C-C chemokines. S5B: violin plot of C-X-C chemokines. S5C: violin plot of interleukin family. S5D: TNF family and other inflammatory factors. ∗ indicates P < 0.05. [file 3558200.f1.zip › Supplemental figure S2B.pdf]

S2C

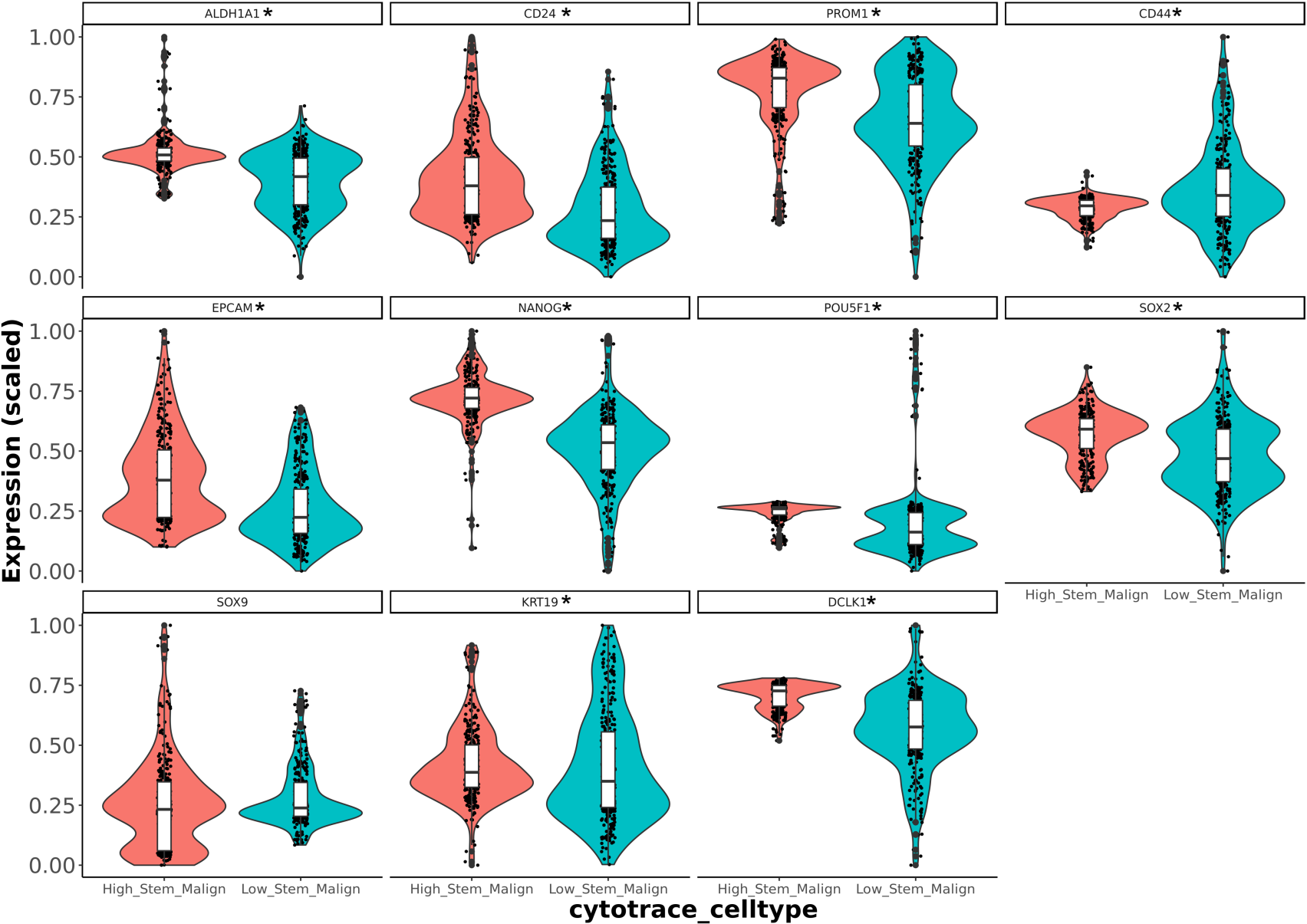

Supplement: Supplementary Materials — Supplemental Table S1: malignant cell counts from 5 tumor samples. Supplemental Table S2: collection of stemness signatures from the previous publications. Supplemental Table S3: fold change of genes between high stemness and low stemness malignant cells and P value from GSE138709. Supplemental Table S4: fold change of genes between high stemness and low stemness malignant cells and P value from GSE125449. Supplemental Figure S1: representative immunofluorescence images. Bar = 37.74 μm. Supplemental Figure S2: differentiation heterogeneity of malignant cells in iCCA from GSE125449. S2A: tSNE plots for malignant cells showing CytoTRACE analysis of malignant cells. S2B: tSNE plots showing the expression of CSC marker genes. S2C: violin plots showing the expression of CSC marker genes. ∗ indicates P < 0.05. Supplemental Figure S3: comparison of TAP1 and TAP2 between high stemness and low stemness iCCA cells from GSE138709, shown with violin plot. ∗ indicates P < 0.05. Supplemental Figure S4: comparison of MHC pathway profile between high stemness and low stemness iCCA cells from GSE125449. S4A: violin plot of MHC I and II pathway-related genes. S4B: violin plot of TAP1 and TAP2. ∗ indicates P < 0.05. Supplemental Figure S5: comparison of inflammatory factors between high stemness and low stemness iCCA cells from GSE125449. S5A: violin plot of C-C chemokines. S5B: violin plot of C-X-C chemokines. S5C: violin plot of interleukin family. S5D: TNF family and other inflammatory factors. ∗ indicates P < 0.05. [file 3558200.f1.zip › Supplemental figure S2C.pdf]

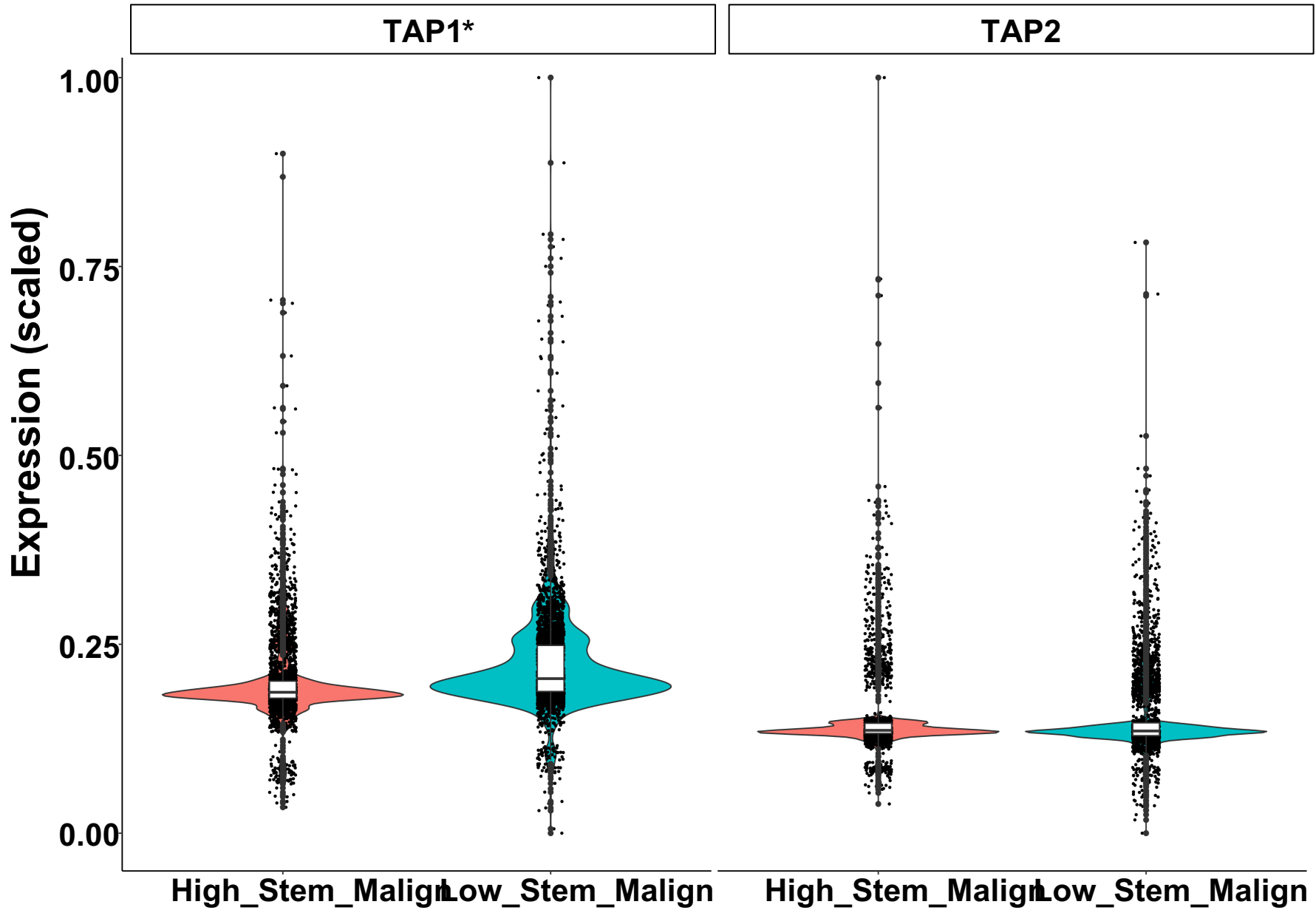

Supplement: Supplementary Materials — Supplemental Table S1: malignant cell counts from 5 tumor samples. Supplemental Table S2: collection of stemness signatures from the previous publications. Supplemental Table S3: fold change of genes between high stemness and low stemness malignant cells and P value from GSE138709. Supplemental Table S4: fold change of genes between high stemness and low stemness malignant cells and P value from GSE125449. Supplemental Figure S1: representative immunofluorescence images. Bar = 37.74 μm. Supplemental Figure S2: differentiation heterogeneity of malignant cells in iCCA from GSE125449. S2A: tSNE plots for malignant cells showing CytoTRACE analysis of malignant cells. S2B: tSNE plots showing the expression of CSC marker genes. S2C: violin plots showing the expression of CSC marker genes. ∗ indicates P < 0.05. Supplemental Figure S3: comparison of TAP1 and TAP2 between high stemness and low stemness iCCA cells from GSE138709, shown with violin plot. ∗ indicates P < 0.05. Supplemental Figure S4: comparison of MHC pathway profile between high stemness and low stemness iCCA cells from GSE125449. S4A: violin plot of MHC I and II pathway-related genes. S4B: violin plot of TAP1 and TAP2. ∗ indicates P < 0.05. Supplemental Figure S5: comparison of inflammatory factors between high stemness and low stemness iCCA cells from GSE125449. S5A: violin plot of C-C chemokines. S5B: violin plot of C-X-C chemokines. S5C: violin plot of interleukin family. S5D: TNF family and other inflammatory factors. ∗ indicates P < 0.05. [file 3558200.f1.zip › Supplemental figure S3.pdf]

# S4A

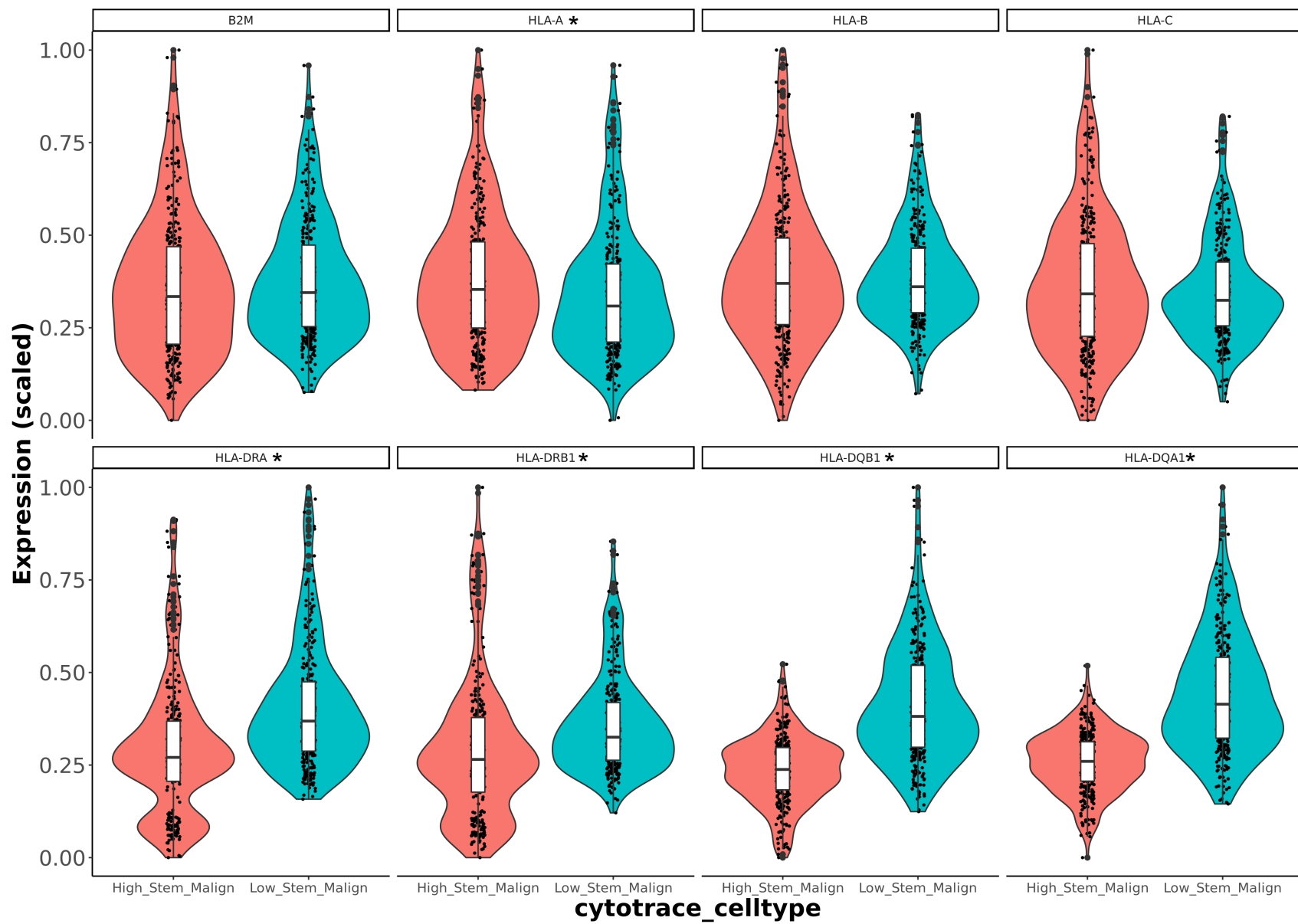

Supplement: Supplementary Materials — Supplemental Table S1: malignant cell counts from 5 tumor samples. Supplemental Table S2: collection of stemness signatures from the previous publications. Supplemental Table S3: fold change of genes between high stemness and low stemness malignant cells and P value from GSE138709. Supplemental Table S4: fold change of genes between high stemness and low stemness malignant cells and P value from GSE125449. Supplemental Figure S1: representative immunofluorescence images. Bar = 37.74 μm. Supplemental Figure S2: differentiation heterogeneity of malignant cells in iCCA from GSE125449. S2A: tSNE plots for malignant cells showing CytoTRACE analysis of malignant cells. S2B: tSNE plots showing the expression of CSC marker genes. S2C: violin plots showing the expression of CSC marker genes. ∗ indicates P < 0.05. Supplemental Figure S3: comparison of TAP1 and TAP2 between high stemness and low stemness iCCA cells from GSE138709, shown with violin plot. ∗ indicates P < 0.05. Supplemental Figure S4: comparison of MHC pathway profile between high stemness and low stemness iCCA cells from GSE125449. S4A: violin plot of MHC I and II pathway-related genes. S4B: violin plot of TAP1 and TAP2. ∗ indicates P < 0.05. Supplemental Figure S5: comparison of inflammatory factors between high stemness and low stemness iCCA cells from GSE125449. S5A: violin plot of C-C chemokines. S5B: violin plot of C-X-C chemokines. S5C: violin plot of interleukin family. S5D: TNF family and other inflammatory factors. ∗ indicates P < 0.05. [file 3558200.f1.zip › Supplemental figure S4A.pdf]

S4B

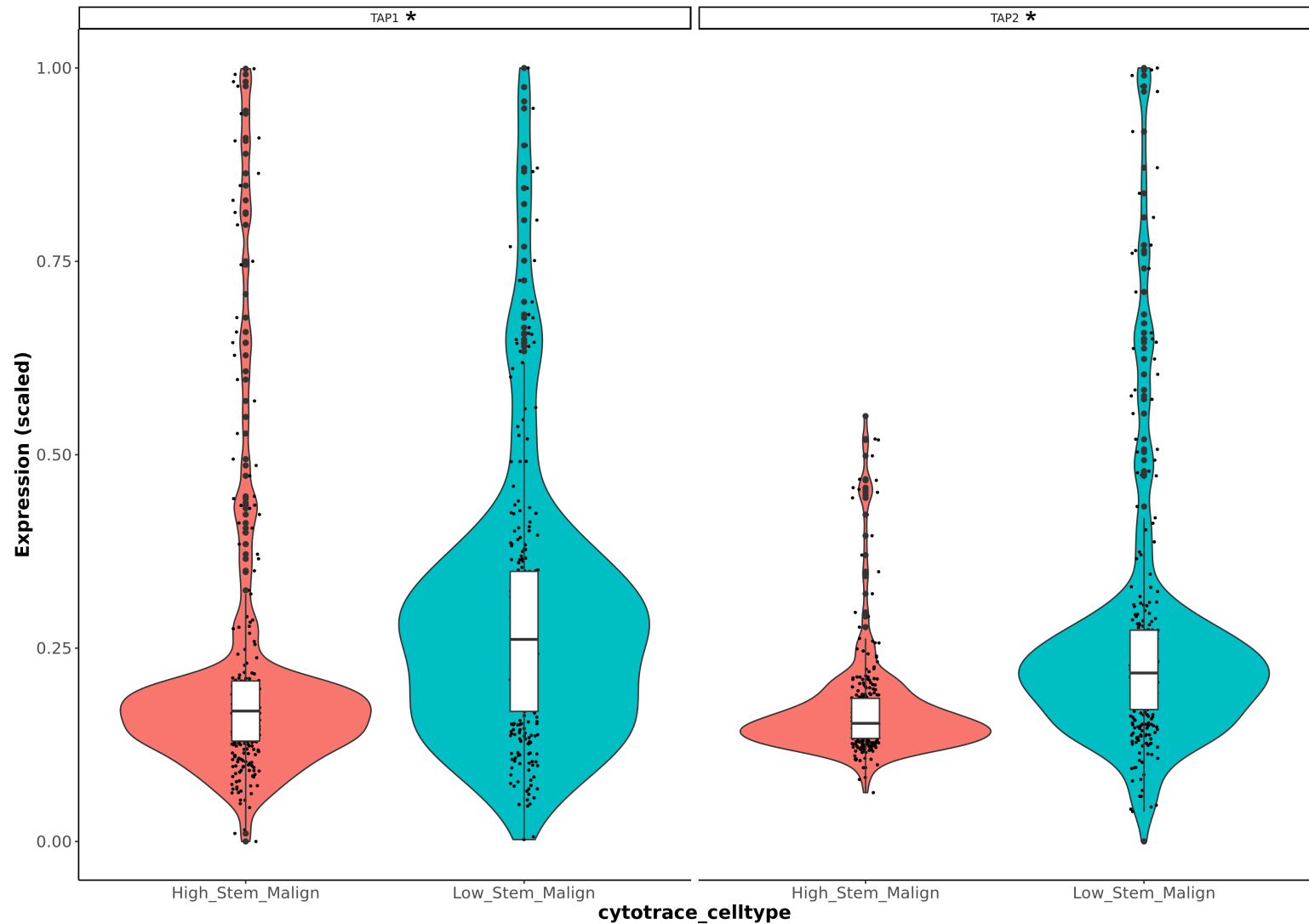

Supplement: Supplementary Materials — Supplemental Table S1: malignant cell counts from 5 tumor samples. Supplemental Table S2: collection of stemness signatures from the previous publications. Supplemental Table S3: fold change of genes between high stemness and low stemness malignant cells and P value from GSE138709. Supplemental Table S4: fold change of genes between high stemness and low stemness malignant cells and P value from GSE125449. Supplemental Figure S1: representative immunofluorescence images. Bar = 37.74 μm. Supplemental Figure S2: differentiation heterogeneity of malignant cells in iCCA from GSE125449. S2A: tSNE plots for malignant cells showing CytoTRACE analysis of malignant cells. S2B: tSNE plots showing the expression of CSC marker genes. S2C: violin plots showing the expression of CSC marker genes. ∗ indicates P < 0.05. Supplemental Figure S3: comparison of TAP1 and TAP2 between high stemness and low stemness iCCA cells from GSE138709, shown with violin plot. ∗ indicates P < 0.05. Supplemental Figure S4: comparison of MHC pathway profile between high stemness and low stemness iCCA cells from GSE125449. S4A: violin plot of MHC I and II pathway-related genes. S4B: violin plot of TAP1 and TAP2. ∗ indicates P < 0.05. Supplemental Figure S5: comparison of inflammatory factors between high stemness and low stemness iCCA cells from GSE125449. S5A: violin plot of C-C chemokines. S5B: violin plot of C-X-C chemokines. S5C: violin plot of interleukin family. S5D: TNF family and other inflammatory factors. ∗ indicates P < 0.05. [file 3558200.f1.zip › Supplemental figure S4B.pdf]

S5A

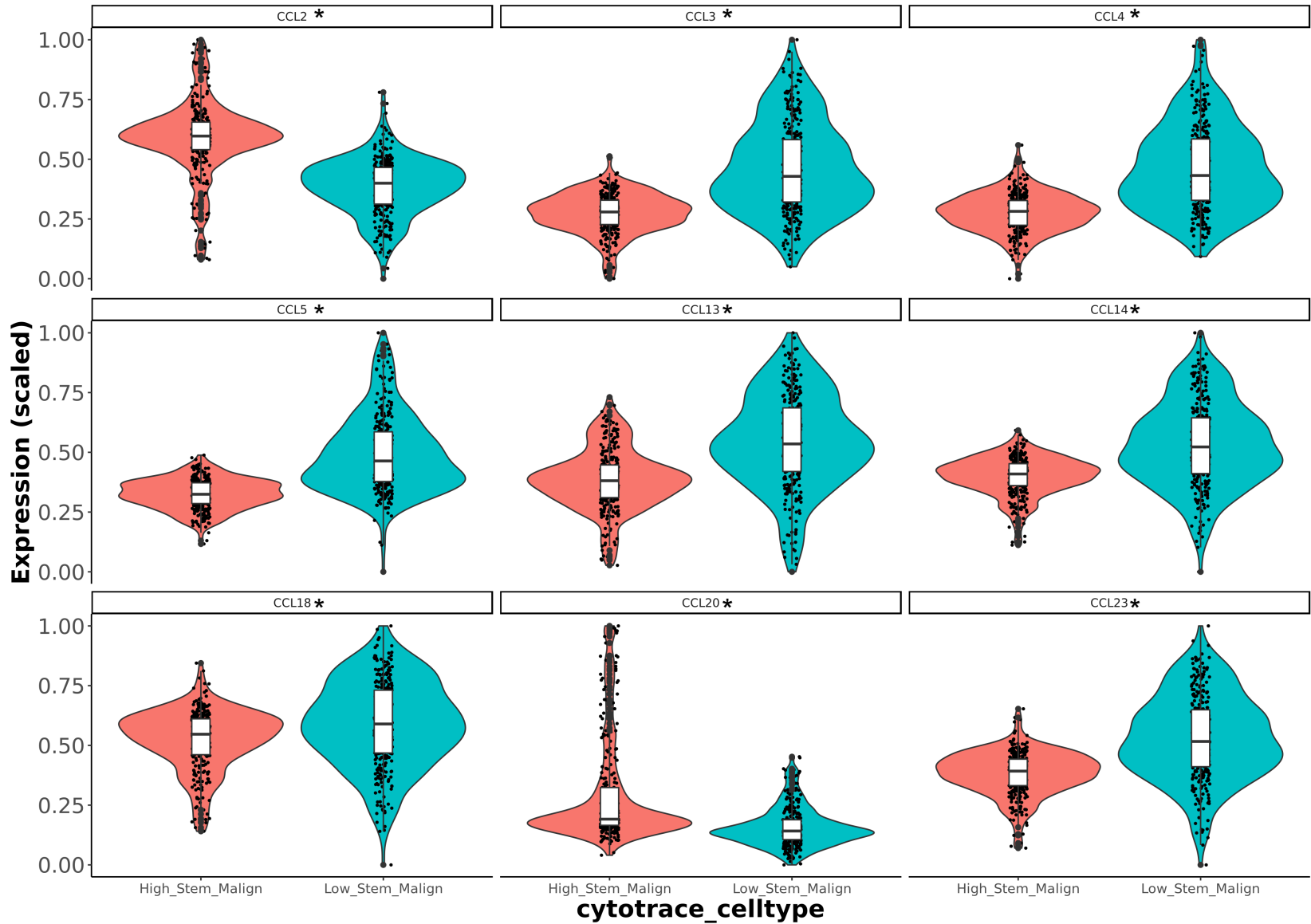

Supplement: Supplementary Materials — Supplemental Table S1: malignant cell counts from 5 tumor samples. Supplemental Table S2: collection of stemness signatures from the previous publications. Supplemental Table S3: fold change of genes between high stemness and low stemness malignant cells and P value from GSE138709. Supplemental Table S4: fold change of genes between high stemness and low stemness malignant cells and P value from GSE125449. Supplemental Figure S1: representative immunofluorescence images. Bar = 37.74 μm. Supplemental Figure S2: differentiation heterogeneity of malignant cells in iCCA from GSE125449. S2A: tSNE plots for malignant cells showing CytoTRACE analysis of malignant cells. S2B: tSNE plots showing the expression of CSC marker genes. S2C: violin plots showing the expression of CSC marker genes. ∗ indicates P < 0.05. Supplemental Figure S3: comparison of TAP1 and TAP2 between high stemness and low stemness iCCA cells from GSE138709, shown with violin plot. ∗ indicates P < 0.05. Supplemental Figure S4: comparison of MHC pathway profile between high stemness and low stemness iCCA cells from GSE125449. S4A: violin plot of MHC I and II pathway-related genes. S4B: violin plot of TAP1 and TAP2. ∗ indicates P < 0.05. Supplemental Figure S5: comparison of inflammatory factors between high stemness and low stemness iCCA cells from GSE125449. S5A: violin plot of C-C chemokines. S5B: violin plot of C-X-C chemokines. S5C: violin plot of interleukin family. S5D: TNF family and other inflammatory factors. ∗ indicates P < 0.05. [file 3558200.f1.zip › Supplemental figure S5A.pdf]

S5B

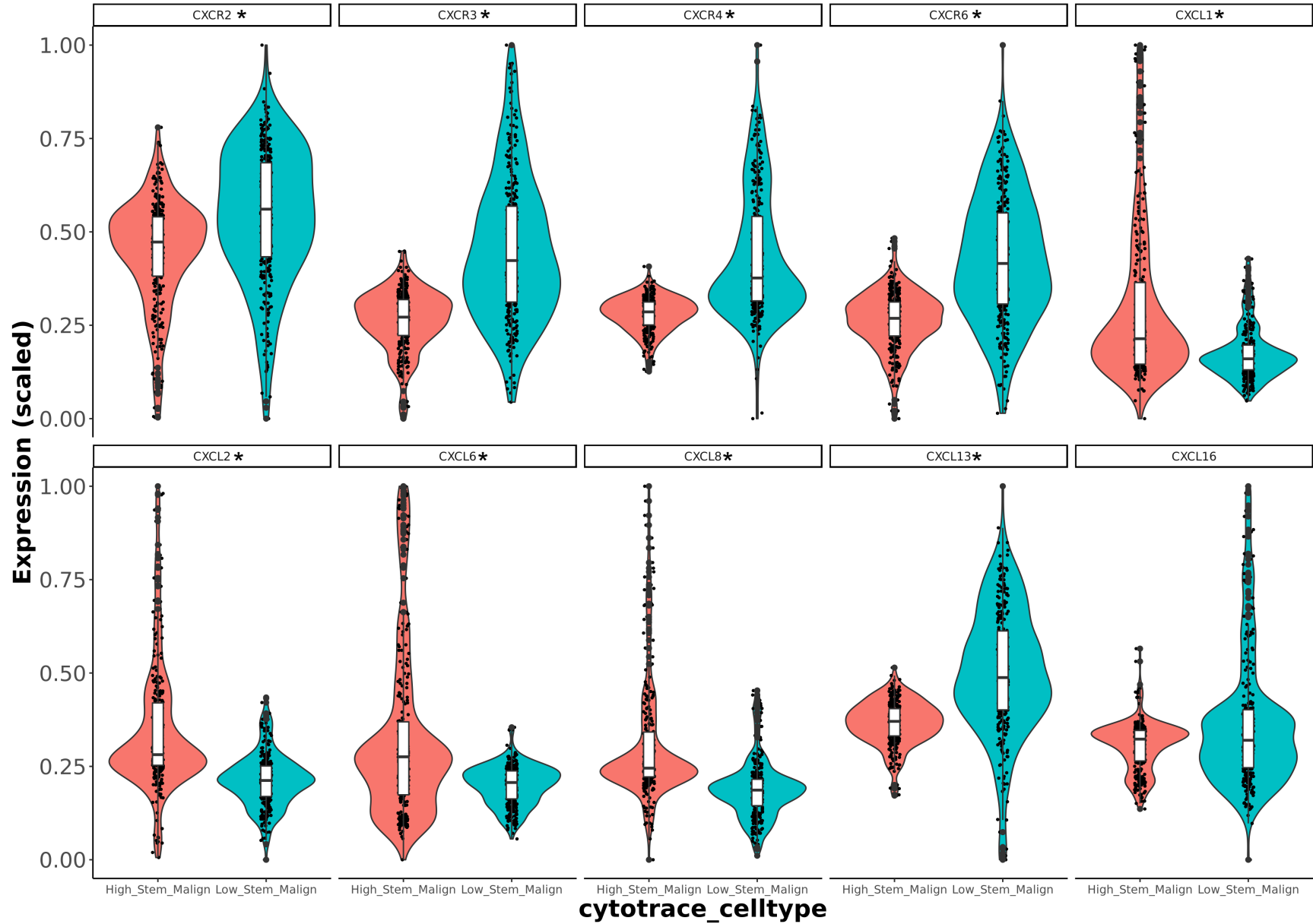

Supplement: Supplementary Materials — Supplemental Table S1: malignant cell counts from 5 tumor samples. Supplemental Table S2: collection of stemness signatures from the previous publications. Supplemental Table S3: fold change of genes between high stemness and low stemness malignant cells and P value from GSE138709. Supplemental Table S4: fold change of genes between high stemness and low stemness malignant cells and P value from GSE125449. Supplemental Figure S1: representative immunofluorescence images. Bar = 37.74 μm. Supplemental Figure S2: differentiation heterogeneity of malignant cells in iCCA from GSE125449. S2A: tSNE plots for malignant cells showing CytoTRACE analysis of malignant cells. S2B: tSNE plots showing the expression of CSC marker genes. S2C: violin plots showing the expression of CSC marker genes. ∗ indicates P < 0.05. Supplemental Figure S3: comparison of TAP1 and TAP2 between high stemness and low stemness iCCA cells from GSE138709, shown with violin plot. ∗ indicates P < 0.05. Supplemental Figure S4: comparison of MHC pathway profile between high stemness and low stemness iCCA cells from GSE125449. S4A: violin plot of MHC I and II pathway-related genes. S4B: violin plot of TAP1 and TAP2. ∗ indicates P < 0.05. Supplemental Figure S5: comparison of inflammatory factors between high stemness and low stemness iCCA cells from GSE125449. S5A: violin plot of C-C chemokines. S5B: violin plot of C-X-C chemokines. S5C: violin plot of interleukin family. S5D: TNF family and other inflammatory factors. ∗ indicates P < 0.05. [file 3558200.f1.zip › Supplemental figure S5B.pdf]

S5C

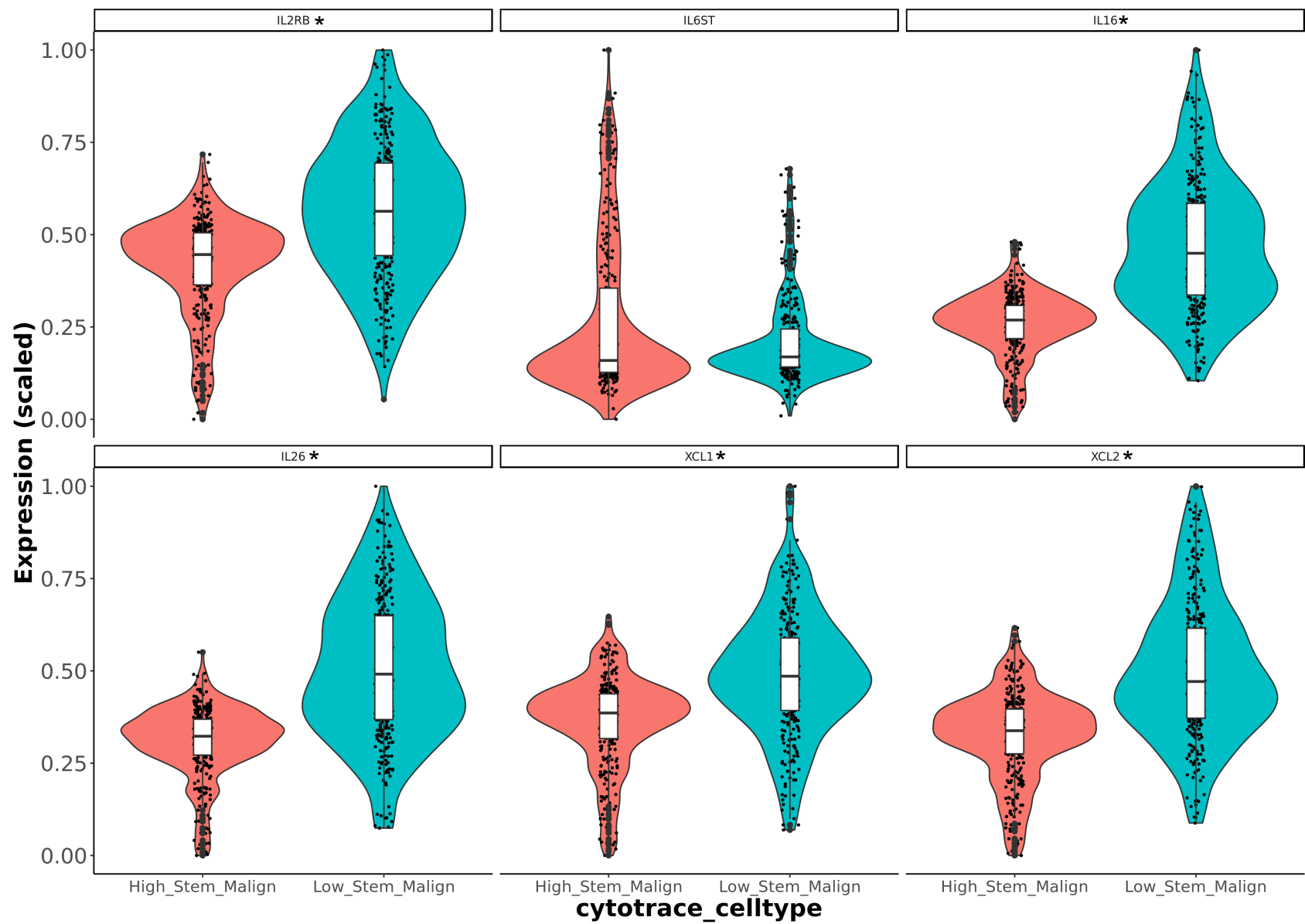

Supplement: Supplementary Materials — Supplemental Table S1: malignant cell counts from 5 tumor samples. Supplemental Table S2: collection of stemness signatures from the previous publications. Supplemental Table S3: fold change of genes between high stemness and low stemness malignant cells and P value from GSE138709. Supplemental Table S4: fold change of genes between high stemness and low stemness malignant cells and P value from GSE125449. Supplemental Figure S1: representative immunofluorescence images. Bar = 37.74 μm. Supplemental Figure S2: differentiation heterogeneity of malignant cells in iCCA from GSE125449. S2A: tSNE plots for malignant cells showing CytoTRACE analysis of malignant cells. S2B: tSNE plots showing the expression of CSC marker genes. S2C: violin plots showing the expression of CSC marker genes. ∗ indicates P < 0.05. Supplemental Figure S3: comparison of TAP1 and TAP2 between high stemness and low stemness iCCA cells from GSE138709, shown with violin plot. ∗ indicates P < 0.05. Supplemental Figure S4: comparison of MHC pathway profile between high stemness and low stemness iCCA cells from GSE125449. S4A: violin plot of MHC I and II pathway-related genes. S4B: violin plot of TAP1 and TAP2. ∗ indicates P < 0.05. Supplemental Figure S5: comparison of inflammatory factors between high stemness and low stemness iCCA cells from GSE125449. S5A: violin plot of C-C chemokines. S5B: violin plot of C-X-C chemokines. S5C: violin plot of interleukin family. S5D: TNF family and other inflammatory factors. ∗ indicates P < 0.05. [file 3558200.f1.zip › Supplemental figure S5C.pdf]

S5D

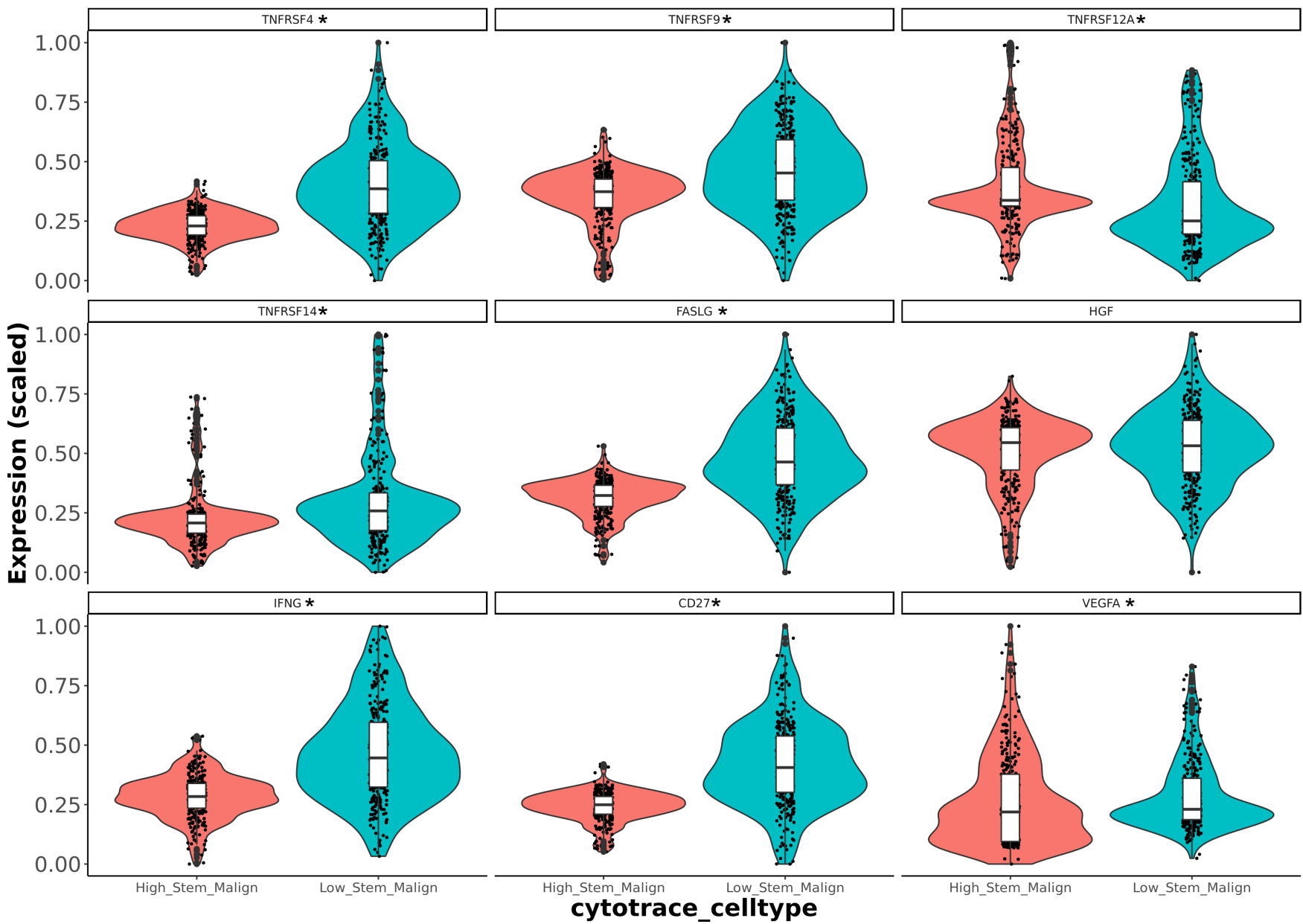

Supplement: Supplementary Materials — Supplemental Table S1: malignant cell counts from 5 tumor samples. Supplemental Table S2: collection of stemness signatures from the previous publications. Supplemental Table S3: fold change of genes between high stemness and low stemness malignant cells and P value from GSE138709. Supplemental Table S4: fold change of genes between high stemness and low stemness malignant cells and P value from GSE125449. Supplemental Figure S1: representative immunofluorescence images. Bar = 37.74 μm. Supplemental Figure S2: differentiation heterogeneity of malignant cells in iCCA from GSE125449. S2A: tSNE plots for malignant cells showing CytoTRACE analysis of malignant cells. S2B: tSNE plots showing the expression of CSC marker genes. S2C: violin plots showing the expression of CSC marker genes. ∗ indicates P < 0.05. Supplemental Figure S3: comparison of TAP1 and TAP2 between high stemness and low stemness iCCA cells from GSE138709, shown with violin plot. ∗ indicates P < 0.05. Supplemental Figure S4: comparison of MHC pathway profile between high stemness and low stemness iCCA cells from GSE125449. S4A: violin plot of MHC I and II pathway-related genes. S4B: violin plot of TAP1 and TAP2. ∗ indicates P < 0.05. Supplemental Figure S5: comparison of inflammatory factors between high stemness and low stemness iCCA cells from GSE125449. S5A: violin plot of C-C chemokines. S5B: violin plot of C-X-C chemokines. S5C: violin plot of interleukin family. S5D: TNF family and other inflammatory factors. ∗ indicates P < 0.05. [file 3558200.f1.zip › Supplemental figure S5D.pdf]
